# Folk standards of sound judgment: Rationality Versus Reasonableness

**DOI:** 10.1126/sciadv.aaz0289

**Published:** 2020-01-08

**Authors:** Igor Grossmann, Richard P. Eibach, Jacklyn Koyama, Qaisar B. Sahi

**Affiliations:** 1 University of Waterloo, 200 University Avenue West, Waterloo, ON N2L 3G1, Canada.; 2 University of Toronto, 100 St. George Street, Toronto, ON M5S 3G3, Canada.; 3 Shaheed Zulfikar Ali Bhutto Institute of Science and Technology (SZABIST), Street # 09, Plot # 67 Sector H-8/4, Islamabad, Pakistan.

## Abstract

Normative theories of judgment either focus on rationality (decontextualized preference maximization) or reasonableness (pragmatic balance of preferences and socially conscious norms). Despite centuries of work on these concepts, a critical question appears overlooked: How do people’s intuitions and behavior align with the concepts of rationality from game theory and reasonableness from legal scholarship? We show that laypeople view rationality as abstract and preference maximizing, simultaneously viewing reasonableness as sensitive to social context, as evidenced in spontaneous descriptions, social perceptions, and linguistic analyses of cultural products (news, soap operas, legal opinions, and Google books). Further, experiments among North Americans and Pakistani bankers, street merchants, and samples engaging in exchange (versus market) economy show that rationality and reasonableness lead people to different conclusions about what constitutes good judgment in Dictator Games, Commons Dilemma, and Prisoner’s Dilemma: Lay rationality is reductionist and instrumental, whereas reasonableness integrates preferences with particulars and moral concerns.

## Teaser

Laypeople grasp economists’ view of rationality, yet favor socially pragmatic reasonableness as a separate standard of judgment.

## INTRODUCTION

What are the key features of a sound judgment? In game theory and dominant streams of economics, sound judgment is intimately linked with the notion of the rational person—a formal, preference-maximizing agent (*1*–*3*). In a disparate literature on political and legal theory, at least since the Roman times, sound judgment has been linked with the well-defined standard of a reasonable person—a pragmatic, socially conscious observer (*4*–*7*). Decades of research in behavioral economics have shown that people often fall short of the rational standard (*8*–*10*), raising the question: How do laypeople understand rationality and do they systematically differentiate it from reasonableness?

If laypeople view rationality and reasonableness as distinct standards of judgment, then deviations of behavior from game theoretical models may not reflect judgment errors (*8*, *10*, *11*), but rather people’s use of a reasonable standard to guide their choices. To explore how people use rationality and reasonableness in their lives, here we use a telescopic approach. First, we examine folk concepts of rational and reasonable actors via content analyses of ascribed characteristics and attributions of personality and behaviors, showing that a rational agent is viewed as a preference maximizer, whereas a reasonable agent is viewed as a satisficer. Moving to implicit norms for reasonable and rational actors, we performed analyses of cultural products (*12*): news, soap operas, legal opinions, and corpora of English, Spanish, Portuguese, and Russian books. We show that linguistic use of “rational” is abstract and individual focused, whereas “reasonable” is context sensitive and socially focused. In subsequent 13 experiments, we demonstrate framing effects of rationality versus reasonableness on expectations and behavior in Commons Dilemma, Dictator Games, and Prisoner’s Dilemma, with expectations for reasonable/rational judgments converging and diverging depending on others’ cooperation- versus competition-signaling behaviors. Last, we replicate the evidence concerning dissociation of rational versus reasonable behavior in a socioeconomically diverse non-Western society.

The distinction between rationality and reasonableness for people’s everyday decisions can be traced back to political and legal theories (see the Supplementary Materials), some claiming that people internalized the chief features of scholarly definitions: rationality as a formal and instrumental standard (*13*), the capacity to exercise judgment in defining one’s key preferences and selecting effective means to pursue those preferences (*14*); reasonableness as a context-dependent and pragmatic standard, balancing realist expectations of most common actions and normative concerns (*15*) in a respectful way (*4*, *16*, *17*).

These theoretical claims remain untested and stand in contrast to a view that in everyday language these concepts appear equivalent. After all, the two terms derive from the same etymological root (*18*). However, if folk understanding of rationality versus reasonableness aligns with behaviors expected from a rational person in game theory and a reasonable person in legal scholarship (*17*, *19*), respectively, then framing decisions in terms of rationality versus reasonableness may prompt people either to maximize their preferences as advocated in neoclassical economics or to flexibly integrate others’ interests as advocated in ethics (*20*–*22*) and political theory (*23*). The chief goal of the present research was to test this possibility.

## RESULTS

### Studies 1 and 2: Personality, stereotypes, and behavioral attributes of rational versus reasonable persons

**Fig. 1:**
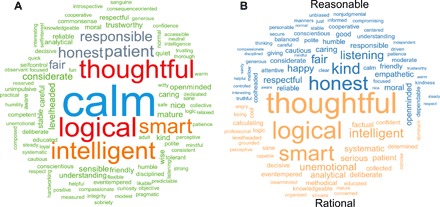
Spontaneous ascriptions of reasonable vs. rational persons. (**A**) Common themes in descriptions of rational and reasonable persons. (**B**) Themes differentiating reasonable (in blue on top) from rational (in brown on the bottom); size of the word is mapped to its maximum deviation across reasonable versus rational text corpora.

In preregistered studies 1 and 2, we examined spontaneous descriptions of people who behave rationally and reasonably and examined attributions of personality characteristics (*24*), societal stereotypes (*25*), and maximizing and satisficing behaviors (*26*) to rational/reasonable actors. As Fig. 1 shows, rational and reasonable were among the most frequent descriptions of each other. The similarities in the descriptors of rational and reasonable actors concerned thoughtfulness, calmness, and intelligence. Consistent with our predictions, we also observed substantial differences. Descriptions of rational persons were more likely to concern abstract and decontextualizing characteristics such as logic, systematicity, analytical skills, and emotional suppression, whereas descriptions of reasonable persons were more likely to include socially conscious characteristics such as honesty, kindness, fairness, and interpersonal sensitivity (also see table S2). We cross-validated these results via hypothesis-blind content analyses, showing that stoic, logic-, and intelligence-oriented characteristics were significantly more likely to be attributed to rational persons (|*Z*s| > 2.84, *P* < 0.005), whereas morality- and interpersonally oriented characteristics were significantly more likely to be attributed to reasonable persons (|*Z*s| > 4.33, *P* < 0.001). There were no significant differences in attributions of levelheadedness and objective/professional characteristics.

**Fig. 2:**
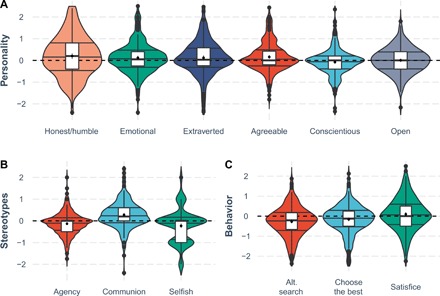
Personality, stereotypes, and behavioral attributes of reasonable vs. rational persons. (**A**) Difference in attribution of personality characteristics to reasonable versus rational persons. (**B**) Difference in cardinal stereotype dimensions of agency and communion ascribed to reasonable versus rational persons, along with ascription of selfishness. (**C**) Expectation of maximizing (alternative search; goal of choosing the best) and satisficing behavior to reasonable versus rational agents. (A) to (C) show violin plots with density distribution of difference scores, 25% median, 75% quantiles, boxplots, estimated means, and bootstrapped 95% confidence intervals. Scores above zero indicate greater attribution to reasonable persons.

Examination of questionnaire-based personality and stereotype-related characteristics yields similar results. As Fig. 2 indicates, participants were significantly more likely to associate the term “reasonable” rather than “rational” with adjectives representing socially oriented characteristics (honesty/humility, agreeableness, emotionality, and extraversion) (2.52 < *t*s < 3.68, 0.012 < *P* < 0.001). Societal stereotypes of rational persons concerned relatively greater agency (*t* = 3.89, *P* < 0.001), whereas stereotypes of reasonable persons concerned greater communion (*t* = 6.70, *P* < 0.0001) and lower selfishness (*t* = 3.77, *P* < 0.001). Moreover, rational persons were viewed as maximizers—pursuing the best option and searching through all alternatives [3.56 < *t*(*df =* 240) < 6.28, *P*s < 0.001], whereas reasonable persons were viewed as satisficers, accepting the best acceptable option [*t*(*df* = 240) = 2.66, *P*s = 0.008].

**Fig. 3:**
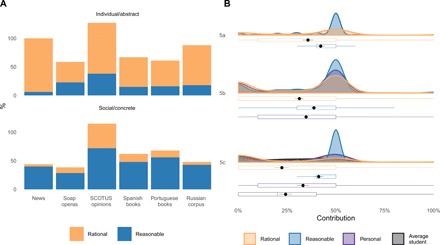
Rationality is associated with individual-focused nouns and rational expectations in a Dictator Game (DG), reflecting preference maximization, whereas reasonableness is associated with socially conscious nouns and reasonable DG expectations, reflecting greater fairness. (**A**) Human-coded percentage of individual/abstract and social/contextual themes in sentences involving “rational” and “reasonable” in text corpora. (**B**) Dictator Game sharing with the other player by reasonable and rational people (studies 5a and 5c), personal sharing (studies 5b and 5c), and an average student (study 5c). Figure represents density distributions, boxplots, and estimated means with 95% confidence interval at α = .05 obtained via bootstrapping with 1000 samples.

As supplementary analyses show, people’s views of rational and reasonable persons were comparatively consistent, whereby the views of a reasonable (versus rational) person were uniquely aligned with attributions of agency, communion, and selfishness to an ideal person. Moreover, characteristics of rational and reasonable persons explained independent variance in attributions of competence to an ideal person. These results both oppose the idea that rationality represents a sole standard of judgment or that reasonableness is a vague, low-relevance concept for judgmental competence (also see consistency of distributions of expected behavior by reasonable versus rational actors in Fig. 3).

### Studies 3 and 4: Computerized and human-coded content analyses of news on the web, American soap operas, SCOTUS opinions, Google books, and national corpora

In study 3, we examined over 5 billion words of data from web-based English language news sources from January 2010 to September 2017—the largest corpus of everyday English language to date. Specifically, we explored the top 100 nouns most frequently following “reasonable” and “rational,” quantifying unique associations and classifying words as abstract/person focused and context sensitive (i.e., variation across either intertemporal or social interpersonal contexts) via human coders. We reasoned that term-specific associations (e.g., idioms) would carry the psychological meaning of the respective constructs. Human-guided content analyses revealed that 33% of nouns following “rational” were classified as abstract/person focused, compared with 6% of nouns following “reasonable” [χ^2^(*df =* 1, *N =* 200) = 23.22, *P* < 0.0001, Cramer’s *V =* 0.341]. Conversely, 40% of nouns following “reasonable” were classified as reflecting contextual contingencies, but only 4% of nouns following “rational” [χ^2^(*df =* 1, *N =* 200) *=* 37.76, *P* < 0.001, Cramer’s *V =* 0.435; see Fig. 3].

We cross-validated our content analyses on soap operas and Supreme Court of the United States (SCOTUS) opinions. In soap operas, compared with 36% of nouns following “rational” classified as abstract/person focused, only 23% of nouns following “reasonable” were classified as abstract/person focused [χ^2^(*df = 1*, *N* = 140) *=* 2.79, *P =* 0.095, Cramer’s *V =* 0.141]. Conversely, 28% of nouns following “reasonable” were classified as reflecting contextual contingencies, whereas only 10% of nouns following “rational” were classified into this category [χ^2^(*df = 1*, *N* = 140) *=* 7.76, *P =* 0.005, Cramer’s *V =* 0.235]. In SCOTUS opinions, compared with 89% of nouns following “rational” classified as abstract/person focused, only 38% of nouns following “reasonable” were classified as abstract/person focused [χ^2^(*df = 1*, *N* = 200) *=* 56.11, *P <* 0.001, Cramer’s *V =* 0.530]. Conversely, 72% of nouns following “reasonable” were classified as reflecting contextual contingencies, whereas only 43% of nouns following “rational” were classified into this category [χ^2^(*df = 1*, *N* = 200) *=* 17.21, *P* < 0.001, Cramer’s *V =* 0.293]. These observations indicate that the notion of reasonableness in cultural products is more likely to take contextual particulars (intertemporal uncertainty and interpersonal considerations) into account, whereas the notion of rationality appears to chiefly focus on the abstract, individual attributes and preferences.

In study 3b, we further bolstered this inference by examining the relative frequencies of definite (“the”) and indefinite (“a”) articles preceding utterances reflecting “reasonable” and “rational” judgment (utterances finishing with “action”/“decision”/“thing to do”). In this context, indefinite articles indicate a general recommendation because they imply that more than one approach could be appropriate (e.g., “a reasonable action”), whereas the definite article indicates the application of a rule because it implies that only one approach is appropriate (e.g., “the rational thing to do”). We examined both the news corpus and the American Google Books—the largest corpus of English books available to date (*27*). In the news, statements reflecting rational judgments were 1.41 times more likely to be preceded by the definite article than statements reflecting reasonable judgments. Conversely, statements reflecting reasonable judgments were 3.84 times more likely to be preceded by the indefinite article than statements reflecting rational judgments. Results were similar for Google books, with definite articles 1.40 times more likely to precede rational judgments and indefinite articles 2.10 times more likely to precede reasonable judgments.

Does the distinction between reasonableness and rationality exist in other languages beyond English? In study 4, we addressed this question by extending human-coded content analyses of the top 100 associations in the Spanish and Portuguese corpora of Google Books, as well as a random subset of 100 sentences including reasonable/rational in the Russian National Corpus (see Supplementary Materials for methods of identifying key terms and their translation). Similar to English, 45 to 70% of nouns following “rational” were classified as individual focused, compared with 15 to 18% of nouns following “reasonable”: Spanish, χ^2^(*df =* 1, *N =* 200) = 30.73, *P* < 0.0001, Cramer’s *V =* 0.392; Portuguese, χ^2^(*df =* 1, *N =* 200) = 19.84, *P* < 0.0001, Cramer’s *V =* 0.315; Russian, χ^2^(*df =* 1, *N =* 200) = 54.87, *P* < 0.0001, Cramer’s *V =* 0.524. Conversely, 43 to 56% of nouns following “reasonable” were classified as reflecting contextual contingencies, whereas only 5 to 14% of nouns following “rational” were classified into this category: Spanish, χ^2^(*df =* 1, *N =* 200) = 27.02, *P* < 0.0001, Cramer’s *V =* 0.368; Portuguese, χ^2^(*df =* 1, *N =* 200) = 43.14, *P* < 0.0001, Cramer’s *V =* 0.464; Russian, χ^2^(*df =* 1, *N =* 200) = 43.14, *P* < 0.0001, Cramer’s *V =* 0.464.

Overall, analyses of written media in English-, Spanish-, Portuguese-, and Russian-speaking countries show qualitatively distinct norms of reasonableness and rationality, such that reasonableness includes interpersonal consideration and focuses on contextual particulars. Conversely, the standard of rationality appears to reflect decontextualized judgments aligned with individual attributes and instrumental preferences.

### Study 5: Expectations for rational and reasonable agents in a Dictator Game

In subsequent experiments, we examined how people differentiate rationality and reasonableness when evaluating choices in the context of a Dictator Game (*9*)—a behavioral economic game where player A can choose what fraction of a resource ($10) to share with anonymous player B, who must accept the offer. If the cultural associations between rationality and individual preference maximization and reasonableness and socially conscious considerations are internalized at the individual level, then we should find that people expect rational choices to be more preference maximizing and reasonable choices to be more socially conscious.

In three experiments, we varied design (within versus between subject), examined different populations (see table S1), and tested several boundary conditions (see Supplementary Materials for more details). In studies 5a and 5b, Amazon Mechanical Turk (MTurk) workers reported expected contributions by reasonable and rational persons in player A’s role. Study 5c replicated effects on university students and explored whether predicted actions for reasonable or rational agents are closer to the perceived typical person in their community and their personal choice as player A.

As Fig. 3 shows, reasonable people were expected to share on average 7 to 20% more than rational people. Reasonable people were expected to share more than rational people: study 5a, *t*(281) = 5.42, η_p_^2^ = 0.095; study 5b, *t*(960.89) = 5.23, η_p_^2^ = 0.027; study 5c, *t*(206) = 8.96, η_p_^2^ = 0.280 (all *P*s < 0.001); a perceived average student: study 5c, *t*(206) = 9.07, η_p_^2^ = 0.290 (*P* < 0.001); or oneself: study 5b, *t*(491) = 3.83, η_p_^2^ = 0.029; study 2c, *t*(206) = 4.95, η_p_^2^ = 0.290 (all *P*s < 0.001). Conversely, average personal sharing was significantly higher than those expected for a rational person [study 5b: *t*(493) = 2.85, *P* = 0.005, η_p_^2^ = 0.016; study 5c: *t*(206) = 5.49, *P* < 0.001, η_p_^2^ = 0.128] or the perceived average student [study 5c: *t*(206) = 5.02, *P* < 0.001, η_p_^2^ = 0.109]. The latter observation dovetails with earlier work on cynical, asymmetric expectations of selfishness by others versus oneself (*28*, *29*).

The observed dissociation was robust to changing scale direction (unreasonable versus irrational) and question form [asking what reasonable/rational people would give versus asking what would be the reasonable/rational amount; (*12*)]. Moreover, self-perceptions of reasonableness and rationality predicted anticipated decisions: Participants who viewed themselves as reasonable shared significantly more (β_Exp5b_ *=* 0.10, *t* = 2.51, *P* = 0.01; β_Exp5c_ *=* 0.33, *t* = 4.96, *P* < 0.001), whereas participants who viewed themselves as rational shared less (β_Exp5b_ *= −*0.06, *t* = 1.49, *P* = 0.14; β_Exp5c_ *=* −0.24, *t* = 3.62, *P* < 0.001).

### Studies 6 and 7: Rational and reasonable personal choice

Two subsequent experiments used between-subject designs and revealed that the distinction between reasonable versus rational agents extends to framing of personal choices (*16*). Participants intended to donate 5% more money in a Dictator Game if they were seeking to be reasonable versus rational (see fig. S2): study 6a, *t*(353.76) = 2.65, *P* = 0.009, η_p_^2^ = 0.016; study 6b, *t*(493.56) = 2.01, *P* = 0.045, η_p_^2^ = 0.008. Study 6b simultaneously tested the impression of selfishness, agency, and communion for rational versus reasonable people. Reasonable people were perceived as less selfish than rational people [*t*(511) = 3.87, *P* < 0.001, η_p_^2^ = 0.028], and this difference in selfishness mediated the difference in predicted sharing by reasonable versus rational people (*Z* = 3.19; 95% CI_bootstrapped_, 0.094 to 344).

Do these effects extend to personal choice on an independent task? In the preregistered study 7 (osf.io/sy24t), participants recalled reasonable or rational actions from their lives and subsequently took part in a standard Dictator Game. Recall of a reasonable action resulted in 3.18% higher offers in the Dictator Game (*M* = $4.28, SD = 1.91) than recall of a rational action (*M* = $3.96, SD = 2.01) [Wald (*df =* 1) = 7.79, *P* = 0.005] (see fig. S3). This effect holds when controlling for socioeconomic factors (age, gender, and income) [Wald (*df =* 1) = 8.21, *P* = 0.004] and with nonparametric analysis of results (*U* = 167,998, *P* = 0.006). Whereas 14.15% of participants in the rational condition donated none of the endowment to the other person, only 9.56% of participants in the reasonable condition suggested donated nothing [*N* = 1116, χ^2^(*df* = 1) = 5.67, *P =* 0.017, Cramer’s *V* = 0.07]. Conversely, 71.01% of participants in the reasonable condition donated at least half of the endowment to the other person, whereas only 65.47% in the rational condition did so [*N* = 1116, χ^2^(*df* = 1) = 5.56, *P =* 0.018, Cramer’s *V* = 0.07].

### Studies 8 and 9: Use of reasonable versus rational standards and generalizability across dilemmas and interpersonal transactions

Study 8 extended the distinction between rational and reasonable standards to expectations in two other classic economic games where a person’s preference conflicts with others’ interests—Commons Dilemma and Prisoner’s Dilemma (*30*). Participants expected rational people to withdraw 12% more from the common pool as compared with reasonable people in the Commons Dilemma [*t*(305) = 5.27, *P* < 0.001, η_p_^2^ = 0.083]. In the Prisoner’s Dilemma, participants expected rational people to pick defecting and cooperative options to a similar extent (defect = 197/cooperate = 190) but expected reasonable people to overwhelmingly select a cooperative option (defect = 84/cooperate = 303) [*N* = 387, χ^2^(*df* = 1) = 7.04, *P* = 0.008, Cramer’s *V* = 0.14].

**Fig. 4:**
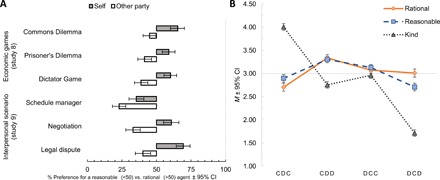
Preference for rational and reasonable agents for self and other across a range of economic and social situations, as well as attributions of rationality, reasonableness, and kindness to cooperating versus defecting behavior in a Prisoner’s Dilemma. (**A**) Rational agent is preferred to act on one’s behalf, and reasonable agent is preferred to act on behalf of another party in studies 8 and 9. (**B**) Attribution of reasonableness is more pragmatic compared with rationality and kindness, as demonstrated by switching in asymmetric multitrial Prisoner’s Dilemma. CDC (prosocial consistency): player A cooperates on round 1, player B defects, and player A continues cooperating on round 2. CDD (punishment): Player A cooperates on round 1, player B defects, and player A defects on round 2. DCC (prosocial switching): player A defects on round 1, player B cooperates, and player A cooperates on round 2. DCD (antisocial consistency): Player A defects on round 1, player B cooperates, and player A continues defecting on round 2. Error bars represent confidence intervals (CIs) at α = .05 obtained via bootstrapping with 1000 samples.

Given the intermediate position of personal choices between rational and reasonable people (Fig. 3), we examined whether participants use the reasonable versus rational choice distinction in economic and interpersonal transactions to their benefit. Specifically, if laypeople internalize both preference-maximizing features of rationality and socially conscious features of reasonableness standards, it is possible that they systematically favor a rational agent in self-focused situations and a reasonable agent in other-focused situations. Study 8 addressed this question in the context of economic games (Dictator Game, Commons Dilemma, and Prisoner’s Dilemma), whereas study 9 extended it to negotiations, legal disputes, and managerial decision-making. For each situation, participants indicated whether they would prefer a reasonable or a rational agent to act on their behalf and on behalf of another party (in a randomized order). In both studies, participants overall favored a rational agent more than a reasonable agent to act on their behalf: study 8, Wald χ^2^(*df* = 1) = 39.40, *P* < 0.001; study 9, Wald χ^2^(*df* = 1) = 7.92, *P* = 0.005 (see Fig. 4). Conversely, participants favored a reasonable over a rational agent for the other parties involved in a dilemma: study 8, Wald χ^2^(*df* = 1) = 21.63, *P* < 0.001; study 9, Wald χ^2^(*df* = 1) = 85.38, *P* < 0.001. See the Supplementary Materials for scenario-specific analyses and results.

### Studies 10 and 11: What actions are rational, reasonable, and kind?

We examined how expectations for reasonable and rational judgments converge and diverge depending on the situation (others’ cooperative versus competitive-signaling behaviors) as a way to further probe whether people view rationality and reasonableness as distinct standards rather than opposite sides of the same evaluative dimension of prosociality. In study 10, participants evaluated reasonableness and rationality of a player in single- and multiround Prisoner’s Dilemmas (see top panel of fig. S4 for types of transactions). In study 11, we rerun the two-round games, simultaneously comparing evaluations of reasonableness to kindness. In single-round games, cooperating players were viewed as more reasonable (versus rational) and defecting players more rational (versus reasonable) [*F*(1,590) = 57.71, *P* < 0.001, η_p_^2^ = 0.089]. In two-round games, when both players behave similarly on round 1 (symmetric game), cooperating on round 2 is viewed as more reasonable and defecting on round 2 is viewed as more rational: study 10, *F*(1,590) = 45.44, *P* < 0.001, η_p_^2^ = 0.072; study 11, *F*(1,733) = 99.01, *P* < 0.001, η_p_^2^ = 0.119. Conversely, under asymmetric conditions of unilateral cooperation by player A on round 1, player A was viewed as equally more rational and reasonable when choosing to defect rather than cooperate on round 2: study 10, *F*(1,590) = 35.18, *P* < 0.001, η_p_^2^ = 0.056; study 11, *F*(1,739) = 35.27, *P* < 0.001, η_p_^2^ = 0.046. Notably, under asymmetric conditions of unilateral defection by player A on round 1, player A was viewed as equally rational when choosing to cooperate or defect on round 2 but was only viewed as reasonable when choosing to cooperate but not defect again on round 2: study 7, *F*(1,589) = 67.84, *P* < 0.001, η_p_^2^ = 0.103; study 11, *F*(1,739) = 82.69, *P* < 0.001, η_p_^2^ = 0.101. The latter observations suggest that depending on the situation, participants considered a given choice as high in rationality and low in reasonableness, low in rationality and high in reasonableness, or as high in both rationality and reasonableness. These results support the idea that people view rationality and reasonableness as distinct standards of judgmental competence rather than merely opposite sides of the same evaluative (prosociality) dimension.

Attributions of reasonableness significantly differed from attributions of kindness: symmetric game, *F*(1,733) = 119.86, *P* < 0.001, η_p_^2^ = 0.141; asymmetric game, *F*(1,739) = 44.17, *P* < 0.001, η_p_^2^ = 0.056. As Fig. 4 indicates, cooperative actions were viewed as kinder rather than more reasonable, whereas reciprocal cooperation after initial defecting and punishment of nonreciprocation after initial cooperation were viewed as reasonable but not kind. Overall, studies 11 and 12 demonstrate that preferences for rational or reasonable agents are conditional on situational goal demands: When choosing an agent that can maximize one’s preferences, people select a rational person, but when choosing an agent that is socially conscious and considerate of other party’s needs, people select a reasonable person.

### Study 12: Spontaneous associations and economic behavior in non-Western, socioeconomically diverse contexts

With an exception of linguistic analyses, our studies have focused on the WEIRD (western, educated, industrialized, rich, and democratic) samples from North America. It is plausible that the thematic associations and economic behaviors would be less pronounced in less-educated, non-Western contexts (*9*, *31*). We examined this question by conducting a preregistered replication of the spontaneous association task (study 1) and the economic expectations in a Dictator Game (study 5) among three social groups in a predominantly collectivist culture, Pakistan: highly educated white-collar workers from the banking sector, less educated street merchants, and rural dwellers mostly reliant on the barter system (versus monetary exchange; see the Supplementary Materials for ethnographic description of each site).

**Fig. 5:**
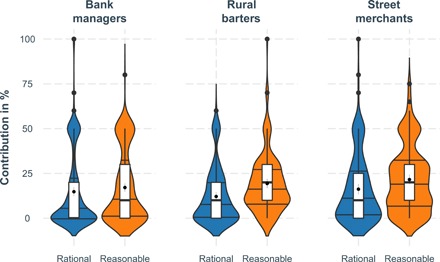
Expected contributions in a Dictator Game by rational and reasonable persons among bank managers, street merchants, and rural barterers in Pakistan. Graphs represent violin plots with density distribution of percentage scores, 25% median, and 75% quantiles, boxplots, estimated means, and bootstrapped 95% confidence intervals.

Similar to study 1, results from human-coded content analyses showed that person-focused attributes were significantly more likely to be mentioned when describing rational persons (|*Z*s| > 2.61, *P* < 0.009), whereas socially conscious characteristics were significantly more frequent when describing reasonable persons (|*Z*s| > 7.12, *P* < 0.0001). Replicating study 5, participants expected reasonable persons to contribute more than rational persons [*t*(609) = 5.10, *P* < 0.0001], with personal choice in-between rational [*t*(609) = 1.96, *P* = 0.050] and reasonable standards [*t*(609) = 3.67, *P* < 0.0001]. As Fig. 5 indicates, differential expectations for reasonable versus rational contributions were especially pronounced among the rural barters [*t*(152) = 4.16 *P* < 0.0001] and street merchants [*t*(168) = 3.73, *P* < 0.001], and less among the urban managers [*t*(287) = 1.76, *P =* 0.075]. Notably, differences in education qualified the rational-reasonable difference [*F*(1,608) = 6.21, *P =* 0.013]: More educated participants were less likely to think that a reasonable person would contribute more and rather be aligned with the notion of preference-maximizing rationality (*B* = −17, 68, SE = 7.48, *t* = 2.37, *P* = 0.018).

## DISCUSSION

It appears that laypeople systematically differentiate rationality and reasonableness along the lines outlined in game theory (*3*, *32*) and legal scholarship (*19*, *33*). A folk standard of rationality chiefly concerns an instrumental focus on individual’s attributes and preferences. In contradistinction, a folk standard of reasonableness concerns a pragmatic focus on social norms and context specificity in the process of judgment (*16*). These views have direct implications for decision-making: Irrational behavior may not necessarily be a sign of failure to understand game theoretical principles but rather an attempt to follow a competing folk standard of reasonableness.

Application of rationality and reasonableness as distinct folk standards of sound judgment appears across five of the most widely spoken languages covering 15% of the world and across affluent and poor populations, including those relying on barter versus monetary economy. Each folk standard can be used to influence decision-making, promoting actions consistent with game theoretical principles in neoclassical economics or actions consistent with the standard of a reasonable person endorsed in legal scholarship and political economy. Evidence for the dissociation between these standards emerges when exploring implicit norms found in human-created cultural products and in behavioral experiments testing social interactions in economic and interpersonal transactions. Further, the distinction between rational and reasonable judgment extends to other aspects of decision-making beyond game theoretical contexts. Specifically, laypeople believe that rationality aligns with a maximizing strategy, whereas reasonableness aligns with satisficing. Thus, rationality and reasonableness capture a relatively broad distinction in laypeople’s standards of judgment. Moreover, although people view reasonable judgments as balancing personal preferences with social norms, they do not do it in a naive, sentimental way—i.e., people think it is reasonable to focus on one’s preferences when others act selfishly.

Our findings oppose the possibility that people fail to understand game theoretical principles likened to standard of rationality in neoclassical economics (*8*). Studies 8 and 9 indicate that people are tactically rational (*34*) when choosing agents to act on their behalf. However, while our results indicate that people may have assimilated the preference-maximizing definition of rationality, this has not crowded out other standards of competent judgment, contrary to concerns raised by some social critics (*32*, *35*, *36*). Humans continue to recognize and apply a distinct standard of reasonableness that balances personal preferences with consideration of others’ interests.

Why do two distinct standards coexist in the same culture and why are they internalized by the same individuals? One of the basic functions of standards is to provide intelligible accounts to justify one’s actions to others. The accountability demands of social life require people to be able to justify their actions, and consensual standards are a useful source of these justifications. Considering social expectations in everyday life (*37*), most people likely internalize both the rational and the reasonable standards because they will face some situations in which they need to justify their preference-maximizing choices and other situations where they need to justify socially conscious choices. Another nonexclusive possibility is that people learn these standards not only to be able to defend themselves but also to keep track of others’ trustworthiness in various contexts (e.g., as partners and as fiduciaries). Last, it is possible that people learn these standards to be better able to hold others into account (*38*). Future work unpacking postchoice interactions between agents involved in a game may help shed light on these theoretical possibilities.

Future research may also explore conditions under which the standard of reasonableness, alone or in conjunction with other ecological factors, contributes to economically irrational choice, and test how egoists and altruists use these standards. Similarly, it is important to explore whether reason-based justifications (*16*) take different forms when people apply rational versus reasonable lay standards. Last, the observation that making the rational (versus reasonable) standard salient can influence people to be preference maximizing (versus socially conscious) also suggests a thus far unexplored intervention to encourage people to make more cooperative choices: reduce the demand to be rational and enhance the request to be reasonable.

## MATERIALS AND METHODS

### Study 1

#### *Participants*

We recruited Amazon MTurk workers who received $1.20 remuneration. Exclusion rates and further demographics are in table S1. As specified in the preregistration protocol (osf.io/af3bw), we aimed to recruit at least 200 participants, oversampling to 250 participants to ensure sufficient power for a within-subject design based on average effect size in social-personality psychology (*r* = 0.21), estimating α/β errors at 5%. This and subsequent human-based studies have been reviewed and received ethics clearance through the University of Waterloo Research Ethics Committee (ORE no. 30580).

#### *Design*

MTurk workers were first presented with the spontaneous adjective ascription task, subsequently evaluated stereotype-content adjectives, followed by the task about similarity of the terms rational-reasonable to adjectives characterizing major personality dimensions. Within each task, the order of presentation (reasonable versus rational) was randomized.

#### *Procedure*

Participants (*N* = 239) were told they would provide their spontaneous thoughts on what are good qualities for decision makers across a variety of situations and answer a few questions about themselves. In the first task, participants were asked to quickly write down the first three characteristics (single words) of people who behave rationally/reasonably. Participants wrote down their answers in the three text boxes.

In the second task, participants were asked to evaluate rational, reasonable, and ideal persons on several different features from the stereotype content questionnaire (communion/warmth: warm, tolerant, good natured, sincere, and trusting; agency/competence: competent, intelligent, confident, independent, competitive), answering the question how they think the society generally views these types of people (1 = not at all to 5 = extremely). Because of special interest in the self-focus, we also included an item concerning being “selfish.” We included ratings for an ideal person to examine alignment with ratings for a rational and reasonable person. This way, we can test normativity and unique contribution of rational/reasonable characteristic with respect to agency and communion. We randomized the presentation of items within each questionnaire. For communion/warmth, we averaged responses to corresponding items (rational, α = .85; reasonable, α = .85; ideal, α = .82). Preliminary principal components analyses (PCA) indicated that “competitive” formed its own component and therefore was not included in the average score of agency/competence (rational, α = .78; reasonable, α = .76; ideal, α = .69). The main analyses remain consistent if including this item.

In the third task concerning personality attributions, participants were told that we are interested in their thoughts on the relationship between the following characteristics to the description of a rational/reasonable person. Participants were instructed to rate how similar is each characteristic to their view of a rational/reasonable person (0 = not at all similar to 10 = extremely similar). Each questionnaire consisted of a set of 10 to 12 adjectives for each of the six themes of the HEXACO personality model (*24*): honesty-humility, emotionality, extraversion, agreeableness, conscientiousness, and openness. We chose HEXACO over the Big 5 due to the addition of the socially conscious honesty-humility dimension, which appeared highly relevant for the purpose of the present investigation. The adjectives were adopted directly from the HEXACO (*24*). To reduce participant burden, we used a missing-at-random procedure, such that participants were presented with a random subset of five adjectives for each of the personality dimensions. We created aggregate average scores for each dimension.

Next, participants rated the extent to which they viewed themselves as reasonable and rational (1 = not at all like me; 2 = not much like me; 3 = somewhat like me; 4 = quite a lot like me, 5 = just like me), provided an open-ended answer to them recalling the nature of the tasks they took part in, and provided demographic information. See materials on Open Science Framework (OSF; osf.io/2h4gx).

#### *Human-guided content analyses*

Two independent, hypothesis-blind coders rated each of the adjectives participants provided on the open-ended initial task for a set of categories determined via a grounded approach—identifying the most frequent themes mentioned in participants’ narratives. Several of these themes concerned individual-focused characteristics (intelligent/smart and self-focused), decontextualized instrumental attributes (stoic and logical/consequential), socially conscious attributes (morals and interpersonal/prosocial), and attributes representing a mixture of individual- and context-focused considerations (objective/professional, personal mastery, and calm/levelheaded). The interrater reliability for each category was very good (Krippendorff’s αs > .89). Minor disagreements (2%) were resolved via a discussion with the first author. We summed participants’ scores across three adjectives they provided.

### Study 2

#### *Participants*

We recruited MTurk workers who received $0.60 remuneration. Exclusion rates and further demographics are in table S1. As specified in the preregistration protocol (osf.io/2x5kb), we aimed to recruit at least 250 participants to ensure sufficient power for a within-subject design based on lowest effect size in study 1 (η^2^ = 0.056), estimating α/β errors at 5%. We oversampled to 280 participants to account for possible attrition due to failure to follow instructions.

#### *Design*

MTurk workers were presented with a task to rate suitability of behaviors for rational and reasonable persons. Within each task, the order of presentation (reasonable versus rational) was randomized.

#### *Procedure*

Participants (*N =* 241) were told they would provide their thoughts on what are good qualities for decision makers across a variety of situations and answer a few questions about themselves. In the two tasks that followed, participants rated whether they thought each of the descriptions fits a characterization of a rational/reasonable person (1 = strongly disagree; 2 = disagree; 3 = somewhat disagree; 4 = neither agree nor disagree; 5 = somewhat agree; 6 = agree; 7 = strongly agree). We specified for each task that we were specifically interested in the participants’ intuitive views of a rational/reasonable person. Items were randomized within each questionnaire.

Behavioral descriptions included items representing two facets of maximizing behavior: goal of choosing the best (eight items; rational, α = .84; reasonable, α = .85) and alternative search (eight items; rational, α = .78; reasonable, α = .80). Further, we included a facet of satisficing behavior (four items representing diagnostic “less ambitious satisficing”; rational, α = .63; reasonable, α = .56). These items and facets were selected on the basis of the most recent recommendations for assessing maximizing and satisficing tendencies (*26*). For a given number of items, the reliability was considered acceptable, with similar results for satisficing when using PCA scores. Following prior work (*26*), we averaged scores for subsequent analyses.

Next, participants rated the extent to which they viewed themselves as reasonable and rational (1 = not at all like me; 2 = not much like me; 3 = somewhat like me; 4 = quite a lot like me; 5 = just like me), provided an open-ended answer to them recalling the nature of the tasks they took part in, and provided demographic information. See materials on OSF (osf.io/2h4gx).

### Study 3a

#### *Corpus selection*

We focused on expressions associated with the words “reasonable” and “rational” in over 5.2 billion words from the NOW (News on the Web) corpus (*39*), which is the largest database of English language newspapers and magazines in the world and is continuously updated. Each night since 2010, automated scripts obtained daily URLs from Google News, downloading 9000 to 10,000 web pages. Texts were stripped of web formatting and tagged and lemmatized for further processing.

#### *Computerized content analysis*

We quantified expressions following “reasonable” and “rational” with the help of computer-guided context-based searchers of collocates (words near a given word). Specifically, we counted all instances in which a noun followed the respective marker within one word apart. This way, we ensured that the noun referred to the “reasonable”/“rational” marker. We examined the top 100 associations as defined by the frequency in the NOW corpus. Because raw frequency is dependent on the size of the all words associated with “reasonable” versus “rational,” we obtained ratio scores of how often a given pair (e.g., rational thought versus reasonable thought) is found in the corpus, which we used as a marker of relative uniqueness. These analyses were obtained using the Now Corpus web interface (corpus.byu.edu/now).

#### *Human-guided content analyses*

Guided by the descriptive evaluation of the unique noun associations with reasonable versus rational markers, we performed a human-guided content analysis. Three independent, hypothesis-blind coders rated each of the top 100 nouns for (i) individual focus, i.e., personal attributes or benefits (e.g., “self” or “person”), and (ii) contextual contingencies, i.e., interpersonal or intertemporal considerations (e.g., “discretion” or “precaution”; see coding manual on the OSF project page), showing very good interrater reliability (self-focus, Krippendorff’s α = .903; contextual contingencies, Krippendorff’s α = .896). Disagreements were resolved by selecting the more common decision among the three raters.

#### *Replication on corpus of soap opera transcripts*

To ensure that our results were not idiosyncratic and specific to the NOW corpus, we sought an additional corpus. Specifically, we explored the prevalence of individual-focused and contingency-related nouns in conjunction with “reasonable” and “rational” in the corpus of 22,000 transcripts from 10 of the most popular U.S. American soap operas from 2001 to 2012 (100 million words; corpus.byu.edu/soap). As this database was significantly smaller, computerized analyses yield 71 nouns following “rational.” Therefore, we restricted this analysis to the top 70 words. As in the main analyses, independent raters coded nouns for individual focus (Krippendorff’s α = .892) and contextual contingencies (Krippendorff’s α = .785). This corpus represents a distinct set of highly popular cultural products, using less formalized English language.

#### *Replication on corpus of SCOTUS opinions*

To ensure that our results were not idiosyncratic and do not merely carry differences between legal and economics vocabulary, we sought a yet additional corpus concerning U.S. Supreme court opinions. Specifically, we explored the prevalence of individual-focused and contingency-related nouns in conjunction with “reasonable” and “rational” in the corpus of 130 million words in 32,000 Supreme Court decisions from the 1790s to March 2017 (corpus.byu.edu/scotus). As in the main analyses, the same independent raters coded nouns for individual focus (Krippendorff’s α = .818) and contextual contingencies (Krippendorff’s α = .567).

### Study 3b

#### *Corpus selection*

We focused on the (i) NOW corpus and (ii) 155 billion n-grams/words of the American literature represented in the largest corpus of books to date (*27*) .

#### *Computerized content analysis*

We selected a range of statements reflecting judgment-related utterances, including “rational [reasonable] thing to do,” “rational [reasonable] decision,” and “rational [reasonable] action” (see the Supplementary Materials for the full list). For each statement, we first obtained raw frequencies when it is preceded by an indefinite article “a” and a definite article “the.” Next, we obtained base rate information for each word/statement (action, decision, thing to do), dividing respective frequency by the base rate. Last, we combined percentages across four statements for comparisons of rational versus reasonable. The pattern of results is comparable when examining individual statements for relative use of indefinite versus definite pronouns.

### Study 4a

#### *Corpus selection*

Following study 3, we focused on the Google books corpora for Spanish and Portuguese languages using the corpus.byu.edu to obtain nouns associated with respective translations of rational and reasonable into Spanish (*racional*/*razonable*) and Portuguese (*racional*/*razoável*). Because in these languages nouns precede these terms, we focused on the top 100 nouns preceding each term. Subsequently, the first author, consulting with native speakers, translated the respective nouns into English for further human-guided coding.

#### *Human-guided content analyses*

As in study 3, two hypothesis-blind coders rated each of the top 100 nouns for (i) individual focus, including personal attributes or benefits (e.g., “self” or “person”), and (ii) contextual contingencies, including interpersonal or intertemporal considerations (e.g., “discretion” or “precaution”). Because some nouns overlapped across corpora, only new words were coded anew (46.75% of the words were not shared with other corpora), with previously established codes used for remaining analyses. These add-on codes showed good interrater reliability (self-focus: Krippendorff’s α = .803; contextual contingencies: Krippendorff’s α = .577). Disagreements were resolved via discussion among the two raters.

### Study 4b

#### *Corpus selection*

Because of the large sampling bias in the Russian Google books sample—all books come from U.S.-based libraries—we sought a different, well-balanced corpus. Specifically, we focused on the Russian National Corpus, created by the Institute of Russian language, Russian Academy of Sciences. The corpus contains 600 million word forms and is well tagged for the present purposes. Because the Russian National Corpus does not provide rankings of the most frequent word associations with the Russian equivalents of the terms “rational” (рациональный)/“reasonable” (разумный), we queried the corpus for a random selection of 100 sentences that include adjectival and adverbial forms of these terms. For adjectives, all three gender forms of respective target terms were included. Further, because pronoun drop is common in Russian and sentence structure is less formalized as compared with English, Spanish, or Portuguese, we included at least three words before/after to provide greater context for each statement. The first author, who is native-level fluent in Russian, translated each statement into Russian, with subsequent human coding performed by independent coders.

#### *Human-guided content analyses*

As in study 3 and study 4a, hypothesis-blind coders rated each of the 100 nouns for (i) individual focus, including personal attributes or benefits (e.g., “self” or “person”) abstract calculations in line with the rational choice theory, and (ii) contextual contingencies, including interpersonal or intertemporal considerations (e.g., “discretion” or “precaution,” “limits,” and “bounds”). Interrater reliability was very good (self-focus: Krippendorff’s α = .930; contextual contingencies: Krippendorff’s α = .931). Disagreements were resolved via discussion with the first author. Main results were very similar when using individual codes by the independent coders.

### Study 5

#### *Participants*

In studies 5a and 5b, we recruited Amazon MTurk workers who received $0.60 remuneration. Participants in study 5c were undergraduate students from the University of Waterloo, Canada, who took part in the study in exchange for a CAD $2 gift certificate. Exclusion rates and further demographics are in table S1. We aimed to recruit at least 130 participants per each of the four between-subject cells in study 5a, following prior recommendations (*40*). Given that the critical factor of study 5a concerned a within-subjects factor (rational versus reasonable), we aimed to double the sample size when examining this factor in a between-subject design in study 5b. Study 5c targeted a more homogeneous sample of nonpsychology major university students. For pragmatic considerations of not extending data collection over one academic term, we aimed to collect as many participants as possible, but at least 110 per between-subject cell. We oversampled target size in each experiment to account for data loss due to noncompliance.

#### *Design*

In studies 5a and 5b, using a within- (study 5a) or a between-subject design (study 5b), Amazon MTurk workers reported expected contributions by reasonable and rational persons in player A’s role. Study 5c replicated effects of the between-subject design on university students and explored whether predicted actions for reasonable or rational agents are closer to the typical student in their community and their personal choice as player A.

#### *Procedure for study 5a*

Participants (*N =* 548) were told they would be reflecting on a situation in an economic game and answering questions about it, as well as answering a few questions about themselves. Participants read a description of an economic game (a Dictator Game, described to participants only as a “game”). Subsequently, they were either asked how much of a resource ($10) would reasonable and rational persons give in player A’s role or what would be reasonable and rational amounts to give in this role. In a different set of experimental conditions, participants were either asked how much of a resource ($10) would unreasonable and irrational persons give in player A’s role or what would be unreasonable and irrational amounts to give. This experimental structure resulted in a 2 (framing: positive versus negative) × 2 (person versus amount) design.

All participants viewed statements in the order specified above (the order of rational and reasonable was counterbalanced in the replication study 5c; see below). Participants wrote a number between 0 and 10 for each characteristic. On the following screen, participants were asked how much they would give to player B if they were in player A’s role. Participants again responded by writing a number between $0 and 10.

Before responding to a final set of demographics items, participants were asked to describe the “money allocation task” (referencing the Dictator Game task) that they had completed in the experiment. Individuals who could not recall any aspect of the task or who left this item blank were screened out before all analyses along with individuals who responded to the Dictator Game task with a number outside the range $0 to 10. All materials are available at the OSF (osf.io/2h4gx).

#### *Procedure for study 5b*

Participants (*N* = 986) read the same game description as in Study 5a (contributions by reasonable and rational people). Next, they were asked to spend some time considering the game instructions before proceeding to bring the main question up on the screen (with the instructions still on the page). Using a between-subject design, we asked participants how much a rational (reasonable) person would give in player A’s role. As in study 5a, this task was followed by a page where participants were asked to indicate how much they would give as player A.

Participants completed a series of filler items. Like in study 5a, the filler task asked participants to complete five words, each with letters missing, by filling in the blanks. This was followed by a 20-item scale of Machiavellianism and a 40-item scale of Rational-Experiential tendencies (*41*). Participants were then asked to rate the extent to which the characteristics reasonable and then rational applied to themselves on a scale of 1 (not at all like me) to 5 (just like me). They were then asked to openly describe the experimental task they took part in, to screen out inattentive participants (see study 1 method), followed by a short page of demographic items. See materials on OSF (osf.io/2h4gx).

#### *Procedure for study 5c*

We conducted a replication of the key conditions of study 5a (positive-person conditions) with an undergraduate university sample (*N* = 207) from the University of Waterloo in Ontario, Canada. Main measures from study 5a were included in study 5c. Participants were presented with the Dictator Game description and on the same page were asked how much a reasonable, rational, and average University of Waterloo student would give in player A’s role. The order of the rational and reasonable items was counterbalanced between participants, and both were presented before the average University of Waterloo student item. As in previous studies, participants were asked how much they would give as player A on the following page. Participants then rated the extent to which the characteristics of reasonable and rational applied to themselves on the same scale as in studies 5a and 5b (1 = not at all to 5 = just like me), and the order of reasonable and rational items was counterbalanced. Last, participants completed a short page of demographic items. See materials on OSF (osf.io/2h4gx).

### Study 6

#### *Participants*

As in studies 5a and 5b, study 6 participants were recruited from MTurk, with $0.60 remuneration. On the basis of study 5 results, we targeted at least 200 participants per condition, which we oversampled to account for data loss due to noncompliance.

#### *Procedure for study 6a*

We used the same between-subject design as in study 5b, but the main question asked participants (*N* = 449) how much they would give as player A if they were trying to be a rational versus a reasonable person. The question that participants saw read as follows: “What would you do if you were in player A’s role and you were trying to be a reasonable [rational] person? Of the $10.00 total, how much would you give to player B in this situation?” After responding to this question, participants filled out a filler task and scales as in study 5b. However, the stimuli in the filler task were updated to contain six new words, followed by the Machiavellianism and Rational-Experiential Scales, the reasonable/rational self-rating scale, and the open-ended screening item (see study 5b method), as well as a short page of demographic items. See materials on OSF (osf.io/2h4gx).

#### *Procedure for study 6b*

We aimed to directly replicate study 6a with additional measures related to folk perceptions of rational or reasonable people in society. Participants (*N* = 515) were asked to consider the Dictator Game instructions and then respond to the same main question as in study 6a. Subsequently, participants were asked why they chose to give player B the amount they did. This task was followed by a page of scale-response items measuring the attribution of selfishness (selfish; 1 = not at all to 5 = extremely) to reasonable versus rational people. As in study 1, we also included supplementary items measuring the attribution to reasonable versus rational people of traits relevant to fundamental dimensions of social perception: agency (competence) and communion (warmth) (*25*, *42*, *43*). There were five items measuring agency (competent, intelligent, confident, independent, and competitive; α = .76), five items measuring communion (warm, tolerant, good natured, sincere, and trusting; α = .85), and one item measuring selfishness (selfish; 1 = not at all to 5 = extremely). See the Supplementary Analyses for more information on these measures and analyses based on these measures. Following the agency, communion, and selfishness measures, participants completed the same screening and demographic items as in previous studies. See materials on OSF (osf.io/2h4gx).

### Study 7

#### *Participants*

Participants were recruited from MTurk, with $0.75 remuneration. On the basis of the study 6 results, we estimated a meta-analytic effect *d* = 0.217 and used a two-tailed test with α = .05. Accordingly, GPower 3.0 suggested a sample size of 1102 to be sufficient for testing the effect in question. On the basis of the exclusion rate in prior studies, we aimed to oversample the target sample by 7%. The final sample and demographics are reported in table S1.

#### *Procedure*

We used a between-subject design consisting of two tasks. In the first task, participants were instructed to recall the last time somebody (themselves or others) acted reasonably (rationally). Participants were asked to try recalling such a situation and describe it in a few sentences in a box provided. As specified in the preregistered exclusion protocol (osf.io/sy24t), we excluded participants who did not complete this task, wrote nonsense, or clearly misunderstood the prompt (i.e., writing focuses on interpersonal transactions broadly without specifying anything related to decision-making, analysis, problem solving, or moral considerations writ large; for example, “I had a pleasant conversation with a friend”). Recall-specific exclusions did not significantly differ by condition (reasonable condition = 4.57% versus rational condition = 5.61%), *N* = 1187, χ^2^(*df =* 1) = 0.961, *P =* 0.327. Next, participants were presented with a standard description of a Dictator Game, asking them to indicate how much money out of $10 they would give to player B (see study 6 methods).

As in prior studies, participants rated the extent to which they viewed themselves as reasonable and rational (1 = not at all like me; 2 = not much like me; 3 = somewhat like me, 4 = quite a lot like me; 5 = just like me) and provided an open-ended answer recalling the nature of the game and provided demographic information. Before debriefing, we asked participants to indicate if they had “any ideas what we were investigating.” As indicated in the preregistered protocol, we used responses in this segment to screen out participants who reported that the study concerned effects of reasonable/rational recall on subsequent choice in the game. Exclusions did not significantly differ by condition (reasonable condition = 1.50% versus rational condition = 1.49%), *N* = 590, χ^2^(*df =* 1) = 1.750, *P =* 0.186.

### Study 8

#### *Participants*

Participants were recruited from MTurk, with $0.60 remuneration. On the basis of earlier studies, we targeted at least 200 participants per condition, which we oversampled to account for data loss due to noncompliance.

#### *Procedure*

We used three economic dilemmas—a Commons Dilemma, a Prisoner’s Dilemma, and a Dictator Game Dilemma, asking participants (*N* = 387) whether they would prefer a reasonable or rational person representing either themselves or the other party in each of the dilemmas. For the first two dilemmas, participants were also asked what move they thought a rational and then a reasonable person would make in the dilemma. Dictator Game instructions were identical to previous studies. Commons Dilemma and Prisoner’s Dilemma were presented as raffle games to avoid familiarity bias. First, participants completed the Commons Dilemma and Prisoner’s Dilemma, each with their associated questions, which were presented in a randomized order to avoid possible order effects. Given the previously established association between the expectations and personal choice for reasonable versus rational agents in a Dictator Game, we aimed to prevent possible carryover effects from expectations in a Dictator Game by examining responses to this dilemma last.

For each dilemma, participants indicated whether they would prefer a reasonable or a rational agent to act on their behalf and on behalf of another party (in a randomized order). To assess expectations for the Commons dilemma, participants indicated their expectation for withdrawal of lottery tickets from a common pool. To evaluate expectations for the Prisoner’s Dilemma, participants indicated whether reasonable/rational agents would choose a prosocial/group-gain maximizing option or rather an individual-gain maximizing option.

Following economic dilemma tasks, participants completed the same measures of agency (α_reasonable_ = .74, α_rational_ = .80), communion (α_reasonable_ = .83, α_rational_ = .83), and selfishness (*25*, *42*) as in study 1, for rational and reasonable people in a randomized order. Participants also responded to the questions about self-rating scale for reasonable then rational characteristics as in study 1 and were asked to recall the three economic games completed earlier as a screening item before filling out a short page of demographics items. See materials on OSF (osf.io/2h4gx).

### Study 9

#### *Participants*

Participants were recruited via MTurk, with $0.60 remuneration. On the basis of earlier studies, we targeted at least 200 participants per condition, which we oversampled to account for data loss due to noncompliance.

#### *Procedure*

Participants (*N* = 291) were told that the aim of this experiment was to determine what were considered good characteristics for decision makers across different scenarios. Participants were presented with a randomized set of seven scenarios. For each scenario, they were asked whether they would choose a rational or reasonable person to fill the described role and why they had made that choice (open-ended response item). Participants evaluated two versions of the question, presented in a randomized order, concerning personal choice and choice for the opposite party involved in the dispute (within-subject component). An example of a script is: “Whom would you prefer to represent your side in a legal dispute—an attorney who has a reputation for being very rational or one who has a reputation for being very reasonable?” In addition to six scenarios analyzed in the main text, we included an exploratory scenario concerning a judge deciding a case one is involved in (see ‘Appendix’ section in the Supplementary Materials). We treated this scenario as exploratory because it did not follow the symmetric set of scenarios benefitting the self- versus other-benefitting choice (i.e., evaluations for the judge are expected to be the same to the self and the other party). Items representing both the participant’s interests and the interests of others were presented. Following each scenario, participants provided an open-ended response explaining their choice (used for screening nonsense responses).

We measured perceptions of agency (α_reasonable_ = .81, α_rational_ = .78), communion (α_reasonable_ = .83, α_rational_ = .84), and selfishness (*25*) for rational and reasonable others (presentation order was randomized) as in study 1. The same filler task and scale for Machiavellianism as in study 6b were presented, followed by the self-rating scale from study 2b and a short page of demographics items. For this experiment, participants were screened on the basis of their response to the seven open-ended scenario items. Participants who provided no response or an incoherent response to these items were screened out. See materials on OSF (osf.io/2h4gx).

### Study 10

#### *Participants*

Participants were recruited via MTurk, with $0.50 remuneration. The study involved randomized presentation of situations involving defecting versus nondefecting players on the first round of the game and separate randomization of situations involving defecting versus nondefecting players on the second round. Therefore, without a prior estimate of effect size, we followed the rule of thumb that we used in prior studies, targeting at least 200 participants per presentation order condition in the first round. We oversampled to account for data loss due to noncompliance.

#### *Procedure*

Participants were told that they would be reflecting on qualities of good decision-making in a variety of situations and their preferences for certain qualities in decision makers. Participants read a description of a variant of a Prisoner’s Dilemma game used in study 8, presented as an anonymous raffle game involving simultaneous exchange between player A and player B, who each had a goal of earning tickets that could be exchanged for a $1000 gift certificate.

Participants were presented with a series of scenarios concerning the two players’ behaviors in the game and were asked to rate how reasonable/rational player A was on a unipolar 1 to 5 scale (1 = not at all; 2 = slightly; 3 = moderately; 4 = very; 5 = extremely). The first two situations, presented in a randomized order, concerned player A choosing either to cooperate or defect on the first round of the game, resulting in a 2 (player A behavior: cooperate versus defect) × 2 (type of judgment: rational versus reasonable) within-subject design. The subsequent set of situations are depicted in Fig. 4. They concerned the behavior of players A and B across two rounds, such that players A and B could cooperate or defect on the first round, and player A could then cooperate or defect on the second round. Again, participants evaluated the rationality and reasonableness of player A’s behavior in each of these scenarios.

We subsequently assessed participants’ stereotypes of rational and reasonable people, similar to prior studies. To this end, after rating these eight scenarios, participants were asked to rate a set of randomized-order characteristics on a bipolar 7-point scale when responding to the question: “As viewed by society, are reasonable or rational people more likely to show the described quality?” (−3 = reasonable persons definitely more likely; −2 = reasonable persons more likely; −1 = reasonable persons slightly more likely; 0 = reasonable and rational persons equally likely; 1 = rational persons slightly more likely; 2 = rational persons more likely; 3 = rational persons definitely more likely). The characteristics were modeled on the dimensions of warmth and competence that we used in earlier studies. Preliminary PCA on the standardized scores indicated that rating of abstract qualities separated into an individual-focused (independent, confident, intelligent, competent, competitive, and self-focused; α = .77) and a socially conscious (warm, good natured, empathetic, sincere, humble, trusting, fair, tolerant, and cooperative; α = .91) component. Therefore, we created composite scores of these components by averaging respective scores.

As in prior studies, participants rated the extent to which they viewed themselves as reasonable and rational (1 = not at all like me; 2 = not much like me; 3 = somewhat like me; 4 = quite a lot like me; 5 = just like me), provided an open-ended answer to them recalling the nature of the game, and provided demographic information.

### Study 11

#### *Participants*

Participants were recruited via MTurk, with $1.20 remuneration. The study was a replication of study 10; thus, we targeted at least 600 participants. We oversampled by 30% to account for data loss due to noncompliance.

#### *Procedure*

The procedure was identical to the two-round game described in study 10. The game involved eight situations, depicting all combination of cooperating versus defecting across two rounds, such that players A and B could cooperate or defect on the first round, and player A could then cooperate or defect on the second round. Participants evaluated the rationality, reasonableness, and kindness of player A’s behavior in each of these scenarios on a 1 to 5 scale (1 = not at all; 2 = slightly; 3 = moderately; 4 = very; 5 = extremely).

As in prior studies, participants rated the extent to which they viewed themselves as reasonable and rational (1 = not at all like me; 2 = not much like me; 3 = somewhat like me; 4 = quite a lot like me; 5 = just like me), provided an open-ended answer to them recalling the nature of the game, and indicated whether they believed there are any reasons why their data should be discarded due to distraction or inattention to the task, and finally provided demographic information.

### Study 12

#### *Participants*

We used services of a professional survey collection company (Neuron Business and Development Solutions) to recruit participants from urban and rural areas in Pakistan. As specified in the preregistration protocol (osf.io/5wfrd), we aimed to recruit at least 600 participants, with even subsamples from each area (see figs. S10 and S11), corresponding to the sample size used in prior studies. We oversampled by 20 participants to account for compliance-based attrition. Half of the participants were recruited on-site in highly educated areas in Islamabad (banks), whereas the other half was split between a subsample of street merchants and another subsample of barter dealers in rural Pakistan (see Supplementary Materials for further details on the data collection sites). Urban participants were compensated 300 rupees (CAD $3). For participants from the sub-urban and rural area, it is considered impolite to receive payment for trivial responses. Therefore, wherever possible/suitable, data collection agents purchased some item(s) from the participants’ businesses to compensate for their efforts.

#### *Design*

Participants were presented with two tasks. In the first task, as in study 5, participants were presented with a Dictator Game. In the second task, as in study 1, participants were asked to use three words to describe a rational person (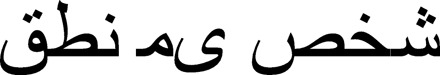) and reasonable person (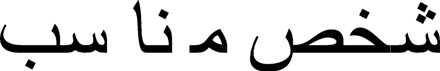). The Urdu words selected for rational and reasonable were determined in consultation with experts in legal and economic scholarship to ensure their representation in local language. The order of presentation (rational versus reasoning) was counterbalanced across participants.

#### *Procedure*

For the urbanized sample, the survey was completed on paper. For the street merchant and rural barter samples, data collection agents administered the survey verbally in the same order. For the first task (Dictator Game with a contribution of 1000 rupees to share), we asked participants to indicate what a rational/reasonable person would do in the role of the player A (dictator). Next, they were asked what they would do in the role of player A. For the second task, we clarified that we particularly looked for adjectives (we avoided to use this word, as noneducated subsamples may not know it) by providing example adjectives that are shared across rational and reasonable persons (“For instance, you can say: ‘A rational/reasonable person is…careful’ or ‘A rational/reasonable person is intelligent’”). Subsequently, participants provided demographic information.

#### *Back-translation*

A bilingual Urdu-English speaker translated all materials into Urdu, and another bilingual translated new materials back into English. The first author discussed the materials with both translators to establish semantic equivalence.

#### *Human-guided content analyses*

Bilingual English-Urdu speakers provided three words to characterize each Urdu word to ensure more precise semantic understanding of the meaning of each word for English-speaking coders. Disagreements between translators were resolved via group discussion. Two independent, hypothesis-blind coders rated each of the words participants provided on the open-ended task for a set of categories determined in study 1 and supplemented with frequently mentioned categories unique to the present sample. Several of these themes concerned person-focused characteristics (intelligent/smart, selfish, and miser), instrumental attributes (logical/consequential), socially conscious attributes (morals, interpersonal/prosocial), as well as attributes representing a mixture of person- and context-focused considerations (objective/professional, personal mastery, and levelheaded). The interrater reliability for each category was very good (Krippendorff’s αs > .84). Very minor disagreements (1%) were resolved via a discussion with the first author. Initial review of open-ended responses indicated that only one-third of participants provided all three words, with the majority of participants providing only one word to describe rational/reasonable persons. This observation is consistent with other research showing lower compliance with repeated questions in non-WEIRD societies (*44*). Therefore, we focused on the analyses of first words. Analyses with percentage of themes per number of completed words indicated very similar results.

## Supplementary Material

http://advances.sciencemag.org/cgi/content/full/6/2/eaaz0289/DC1

Download PDF

Folk standards of sound judgment: Rationality Versus Reasonablenes

## SUPPLEMENTARY MATERIALS

Supplementary material for this article is available at http://advances.sciencemag.org/cgi/content/full/6/2/eaaz0289/DC1 Supplementary Methods Supplementary Analyses Alternative accounts of the present findings Fig. S1. Word clouds of the top 100 nouns following “rational” and “reasonable” in English language newspapers and magazines. Fig. S2. Participants’ contributions in the Dictator Game as reasonable versus rational agents in study 6. Fig. S3. Distribution of donations in the Dictator Game after reminders of rational versus reasonable experiences in study 7. Fig. S4. Attribution of reasonableness and rationality to player A in multiround Prisoner’s Dilemma in studies 10 and 11. Fig. S5. Attribution of reasonableness and rationality to player A in a single-shot Prisoner’s Dilemma in study 10. Fig. S6. Attribution of reasonableness and rationality in study 10 for cooperating and defecting players on a second round of Prisoner’s Dilemmas after bilateral cooperation in the first round. Fig. S7. Attribution of reasonableness and rationality in study 10 for cooperating and defecting players on a second round of Prisoner’s Dilemmas after unilateral cooperation in the first round. Fig. S8. Attribution of reasonableness and rationality in study 10 for cooperating and defecting players on a second round of Prisoner’s Dilemmas after unilateral defecting in the first round. Fig. S9. Attribution of reasonableness versus rationality to different behavioral characteristics to a rational and reasonable person in study 10. Fig. S10. Photographic depiction of survey collection sites in study 12. Fig. S11. Typical institutions and common places for each of the three data collection sites in Pakistan in study 12. Table S1. Demographic information for samples used across experiments. Table S2. Most frequent words (top 10%) when describing rational and reasonable persons. Appendix References (*45*–*53*)
