# Supplementary material for: Folk standards of sound judgment: Rationality Versus Reasonableness
Source: Sci Adv. 2020 Jan 8;6(2):eaaz0289. doi: 10.1126/sciadv.aaz0289 (PMC6949030; doi:10.1126/sciadv.aaz0289)
Supplement: http://advances.sciencemag.org/cgi/content/full/6/2/eaaz0289/DC1 [file supp_6_2_eaaz0289__index.html]

Science Advances | Science AdvancesAAASSearchScience AdvancesMenu

## Supplementary Materials

**This PDF file includes:**

- Supplementary Methods
- Supplementary Analyses
- Alternative accounts of the present findings
- Fig. S1. Word clouds of the top 100 nouns following “rational” and “reasonable” in English language newspapers and magazines.
- Fig. S2. Participants’ contributions in the Dictator Game as reasonable versus rational agents in study 6.
- Fig. S3. Distribution of donations in the Dictator Game after reminders of rational versus reasonable experiences in study 7.
- Fig. S4. Attribution of reasonableness and rationality to player A in multiround Prisoner’s Dilemma in studies 10 and 11.
- Fig. S5. Attribution of reasonableness and rationality to player A in a single-shot Prisoner’s Dilemma in study 10.
- Fig. S6. Attribution of reasonableness and rationality in study 10 for cooperating and defecting players on a second round of Prisoner’s Dilemmas after bilateral cooperation in the first round.
- Fig. S7. Attribution of reasonableness and rationality in study 10 for cooperating and defecting players on a second round of Prisoner’s Dilemmas after unilateral cooperation in the first round.
- Fig. S8. Attribution of reasonableness and rationality in study 10 for cooperating and defecting players on a second round of Prisoner’s Dilemmas after unilateral defecting in the first round.
- Fig. S9. Attribution of reasonableness versus rationality to different behavioral characteristics to a rational and reasonable person in study 10.
- Fig. S10. Photographic depiction of survey collection sites in study 12.
- Fig. S11. Typical institutions and common places for each of the three data collection sites in Pakistan in study 12.
- Table S1. Demographic information for samples used across experiments.
- Table S2. Most frequent words (top 10%) when describing rational and reasonable persons.
- Appendix
- References (*45*–*53*)

Download PDF

**Files in this Data Supplement:**

- Adobe PDF - aaz0289\_SM.pdf
