# Supplementary material for: Folk standards of sound judgment: Rationality Versus Reasonableness
Source: Sci Adv. 2020 Jan 8;6(2):eaaz0289. doi: 10.1126/sciadv.aaz0289 (PMC6949030; doi:10.1126/sciadv.aaz0289)
Supplement: Download PDF [file aaz0289_SM.pdf]

Science Advances
AAAS

advances.sciencemag.org/cgi/content/full/6/2/eaaz0289/DC1

Supplementary Materials for

Folk standards of sound judgment: Rationality Versus Reasonableness

Igor Grossmann*, Richard P. Eibach, Jacklyn Koyama, Qaisar B. Sahi

*Corresponding author. Email: igrossma@uwaterloo.ca

Published 8 January 2020, *Sci. Adv.* **6**, eaaz0289 (2020)
DOI: 10.1126/sciadv.aaz0289

This PDF file includes:

Supplementary Methods
Supplementary Analyses
Alternative accounts of the present findings
Fig. S1. Word clouds of the top 100 nouns following “rational” and “reasonable” in English language newspapers and magazines.
Fig. S2. Participants’ contributions in the Dictator Game as reasonable versus rational agents in study 6.
Fig. S3. Distribution of donations in the Dictator Game after reminders of rational versus reasonable experiences in study 7.
Fig. S4. Attribution of reasonableness and rationality to player A in multiround Prisoner’s Dilemma in studies 10 and 11.
Fig. S5. Attribution of reasonableness and rationality to player A in a single-shot Prisoner’s Dilemma in study 10.
Fig. S6. Attribution of reasonableness and rationality in study 10 for cooperating and defecting players on a second round of Prisoner’s Dilemmas after bilateral cooperation in the first round.
Fig. S7. Attribution of reasonableness and rationality in study 10 for cooperating and defecting players on a second round of Prisoner’s Dilemmas after unilateral cooperation in the first round.
Fig. S8. Attribution of reasonableness and rationality in study 10 for cooperating and defecting players on a second round of Prisoner’s Dilemmas after unilateral defecting in the first round.
Fig. S9. Attribution of reasonableness versus rationality to different behavioral characteristics to a rational and reasonable person in study 10.
Fig. S10. Photographic depiction of survey collection sites in study 12.
Fig. S11. Typical institutions and common places for each of the three data collection sites in Pakistan in study 12.
Table S1. Demographic information for samples used across experiments.
Table S2. Most frequent words (top 10%) when describing rational and reasonable persons.
Appendix
References (*45–53*)

Supplementary Methods

Representative theoretical positions on rationality and reasonableness

The theoretical distinction between rationality and reasonableness is pronounced in ethics and political theory (*7, 19, 45*). For instance, John Rawls defines rationality similar to neoclassical economists, writing that a rational person exercises "powers of judgment and deliberation" both in "seeking ends and interests peculiarly its own" and in "adopt[ing] the most efficient means to ends" (p. 50). In this theoretical framework, rationality is a critical capacity because it allows people to define and pursue their interests. However, on its own rationality lacks a concern for interests of other people and broader moral concerns contributing to a fair society. Further, Rawls defines reasonableness as the capacity to exercise judgment to take into account others' interests and balance those interests with one's own in a mutually respectful way. Rawls (*46*) writes, "[Persons] are reasonable when... they are prepared to offer one another fair terms of social cooperation... and they agree to act on those terms, even at the cost of their own interests in particular situations" (p. 136). Thus, Rawls (*14*) indicates that, unlike rationality, reasonableness is "incompatible with egoism" (p. 49).

In other words, whereas the reasonable standard is intimately linked to the idea of consideration of others' interests and viewpoints, to give them proper due, the rational standard may or may take others' interests into account (*33*). Defined this way, both rationality and reasonableness are distinguishable from deontological principles or moral concepts such as courage, kindness, or temperance. For instance, the standard of a reasonable person can be interpreted both from a perspective of a consequentialist cost-benefit efficiency, a deontological perspective of fairness as a principle(*33*). Also, in contrast to the utilitarian principle of aggregate well-being as a sole moral value, rationality and reasonableness allow a variety of personal moral reasons (*47*).

As capacities to exercise judgment, rationality and reasonableness can be viewed as *practical* concepts, with rationality oriented toward the individual's goals -- identifying and pursuing one's preferences, and discounting seemingly unnecessary particulars -- and reasonableness oriented toward the interpersonal particulars even if they go against one's immediate preferences – discerning how to apply specific moral virtues in the process of making decisions. Both standards are necessary because a sound decision-maker both needs to understand how to maximize their preferences and also understand how to balance consideration of others' preferences (*48*).

Conceptually related arguments have been consistently made in sociology, philosophy, and political economy throughout the 19th 20th, and early 21st centuries. These concepts vary in nuances (e.g., the importance of pragmatism) but nevertheless show substantial overlap in meaning, as indicated in the table below. Moreover, several of the theorists including Weber highlight the need to balance both principles for a sound judgment.

	**Rationality**	**Reasonableness**
Max Weber (*13*)	Instrumental rationality (Zweckrational)	Value-based rationality (Wertrational)
Jürgen Habermas (*13, 49*)	Instrumental rationality	Communicative action
Robert Nozick (*50*)	Causally expected utility and evidentially expected utility	Symbolic utility
Steven Toulmin (*4*)	Abstract, formal, decontextualized	Informal, pragmatic, context-sensitive

Study 1: *Computerized content analyses*

To supplement human-based analyses, the same adjectives were converted into corpora of rational and reasonable words. In the preprocessing stage, for participants who wrote sentences instead of adjectives (< 5%), responses were converted into common adjectives. Subsequently, we created frequency tables of common and distinct adjectives in each corpus, allowing for analyses of distinct and overlapping words, as well as percentages of most frequent (top 10%) words in the reasonable and rational corpora.

Study 1: exploratory measures

For exploratory purposes participants evaluated how "grounded" they perceived rational/reasonable person. Another exploratory condition involved a similar questionnaire concerning stereotype evaluations of a "normal person."

Study 3a: additional corpus information

News sources included newspapers such as *The Wall Street Journal, The Daily Mail*, *The Financial Times*, and *The Globe and Mail* as well as online and radio news such as *The Huffington Post*, and *National Public Radio*. The corpus was based on a wide range of countries, with over 70% from the U.S. (18.36%), Great Britain (14.75%), Canada (14.09%), Australia (7.71%), Ireland (6.69%), South Africa (5.79%), and New Zeeland (3.36%).

Study 3b: List of judgment-related utterances for definite/indefinite articles

We focused on three key utterances reflecting judgments: "reasonable/rational action," "reasonable/rational decision," and "reasonable/rational thing to do." Here, we specifically targeted action-oriented prompts, rather than subsequent evaluation of the decisions. Therefore, we did not include statements such as "reasonable/rational judgment" and "reasonable/rational outcome." The latter utterances tend to be used retrospectively and therefore introduce unnecessary confounds.

Study 4a-b: Selection of key terms

Reasonable and rational share the same latin root (ratio). The English version of "rational" originates from Old French racionel and directly from Latin rationalis "of or belonging to reason,

reasonable," which has its root in ratio (genitive rationis) "reckoning, calculation, reason." Similarly, the English version of "reasonable" originates from Old French raisonable, which in turn originates from ratio "reckoning, understanding, motive, cause." Given that both Spanish and Portuguese are Romance languages (Spanish: racional/razonable; Portuguese: racional/razoável), the same distinction exists in these languages and neatly maps on the terms used in English today.

To cross-validate semantic equivalence of the meaning of each term, we utilized Google Translate (translate.google.com), which utilizes a state-of-the-art neural machine translation engine that looks for contextual patterns trained on hundreds of millions of UN and European Parliament multi-language transcripts to help decide on the best translation. Specifically, we first translated the contextualized terms "rational/reasonable person/action" into respective languages. Next, we examined Google Translate to examine suggested synonyms for the back-translated terms, to ensure they map on the semantic meaning of the original English terms.

In Study 4b, The Russian word for "rational" stems from the same etymological Latin root as its English counterpart. In contrast, the translation for "reasonable" (разумный) has "разум" (mind, reason) as a stem. We used the same Google Translate statistical cross-validation procedure as in Study 3, selecting the terms based on the semantic meaning of the suggested synonyms of each term.

Study 5: Exploratory measures

Given the exploratory nature of this experiment, two additional characteristics were displayed to each participant after reasonable and rational: *wise* and *intelligent* (positive characteristic condition) vs. *unwise* and *unintelligent* (negative characteristic condition). These variables were not included in analyses, as they followed the initial set of reasonable and rational questions.

Further, after a filler task, participants completed a set of exploratory scales which lasted roughly ten minutes (see Supplementary Appendix 1). This filler task was used to avoid effects of specific responses on a previous task on subsequent answers. Specifically, the filler task was designed to divert attention by instructing participants to fill in letters for a series of words. By diverting attention we aimed to attenuate priming effects (*51*). Subsequently, participants rated the extent to which each of the characteristics seen previously (rational, reasonable, wise, intelligent) applied to them on a 1=*Not at all like me* to 5=*Just like me* scale. Analyses related to these self-ratings had to be excluded from Study 5a results because a typo in the materials made this measure invalid.

Study 10: Exploratory measures

For exploratory purposes, participants subsequently filled out a novel scale examining specific behavioral attributes of rational and reasonable persons, which we designed to zero in "focus on social context," "recognition of uncertainty," "fairness-based punishment," as well as "de-contextualized self-focus" (see supplementary fig. S9 for exact items and descriptives for each score). The results on these exploratory items mirrored the pattern of results presented on trait-scores in the main body of the text.

Study 12: Sample site characterization

The survey company collected data at three distinct sites, focusing on white-collar employees in the banking sector in the urban centers ($M_{age=}$30.35; *SD*=6.61; 74%$_{male}$; $M_{years\ of\ education=}$15.85; *SD*=1.35), uneducated poor from the sub-urban market areas around Islamabad ($M_{age=}$33.77; *SD*=8.92; 100%$_{male}$; $M_{years\ of\ education=}$5; *SD*=3.67), and the barter dealers from the rural areas ($M_{age=}$36.22; *SD*=8.66; 100%$_{male}$; $M_{years\ of\ education=}$3.5; *SD*=3.45). The gender specific demographics indicate that the sample was predominantly make, especially in the low-educated areas. This is partially because both street merchants and rural farmers and craftsmen in Pakistan tend to be male, as well as because interviewers were male, making it challenging to recruit females in a predominantly Muslim environment. Most frequent occupations of participants from the urban sub-sample were banker (16%), business development officer (6.3%), loan officer (6.3%), cashier (5.9%), operations manager (5.9%), product manager (5.6%), and customer support officer (5.2%). All other participants from this sub-sample were also from the middle and upper-level management of the banking sector. Most frequent occupations of participants from the street merchant sub-sample were street cart vendor (32%), vegetable/fruits vendor (38.5%), snacks vendor (15.4%), and daily supplies vendor (3%). Most frequent occupations of participants from the rural barter sub-sample were farmers (17%), clay crafter (11.8%), household worker (16.3%), and cobbler (8.5%). Below, we provide a brief description of each data collection site. See fig. S10 for photographic depiction of each site, as well as fig S11 for sample pictorials of key institutions from each site.

Bankers. Respondents included from the metro towns of Islamabad, Rawalpindi, Lahore and Faisalabad. These metro towns are among the top ten most populous cities of Pakistan. Bankers were contacted by visiting their respective branches/offices. Branches were selected from the main commercial areas of the cities.

Street Merchants. Street merchants were approached in the sub-urban areas of Miani, Mandi Bahaudin and Bhara Kahu. These areas have low to middle income class population with relatively higher literacy rate and diversity of professions. Primary income generation activities include agriculture, small to medium businesses and jobs. Data collection agents were advised to start walking from a prominent landmark (e.g., Government Building, Mosque) towards one direction and collecting data from every 3rd street merchant along the way.

Barter System – Villages. Historically, traditional economy in the rural areas have been woven on each other’s dependency leading towards barter system. This barter system still survives – to some extent – in rural areas where dependency on the modern currency is still not absolute. Respondents for this category were selected from the villages and surrounding Moza’s (a sub-unit of village) of the Ghughat and Sigh Bala from Sargodha District, as well as from Kadhar Sharif of Mandi Bahauddin District. Villages are home to majority of the low-income residents, but also include some middle-income residents. Majority of the population rely on agriculture and related professions. Literacy rate is also relatively low compared to the urban population. Respondents were approached by visiting their places in the villages. They were asked whether they deal in barter before collecting their responses.

Supplementary Analyses

Study 1

Computerized analysis of most frequent words in rational/reasonable corpora. Table S2 shows most frequent words used to describe rational and reasonable persons. Similar to the results of the human-coded analyses, results from the computerized analyses first indicate an overlap, with words such as thoughtful, calm, honest, fair, patient, logical, intelligent and smart appearing in both lists. Furthermore, rational was among the most frequent words when describing a reasonable person and vice versa. At the same time, there were substantial differences. Consistent with the human-coded analyses and our prediction, words such as analytical, systematic, unemotional, factual and decisive uniquely appearing in the descriptions of a rational person, consistent with the view of a rational person as an instrumental, calculating agent. Conversely, words such as listening, understanding, respectful, open-minded, moral, caring, kind and friendly appearing when describing a reasonable person, consistent with the view of a reasonable person as a socially-conscious agent. Moreover, among the common words, logical and thoughtful was twice as frequent for rational vs. reasonable person, whereas honest and fair were more frequent for reasonable vs. rational person.

Consistency of stereotypes for rational vs. reasonable persons. *PCA*s of stereotype content adjectives has shown comparable % variance explained by agency (rational: 16.91; reasonable: 15.52) and communion components (rational: 45.78; reasonable: 46.26), with similar association between these components (rational r=.39; reasonable r=.43). This evidence of measurement equivalence for both rationality and reasonableness allows for further interpretation of mean-level differences between ratings of agency and communion for each standard.

Are stereotypes for rational and reasonable persons comparable to those of an ideal person? We addressed this question by running a series of OLS regressions with participants' ratings stereotype content ratings for rational and reasonable persons predicting ratings for an ideal person. For agency, both rational and reasonable scores uniquely contributed to stereotype ascription for an ideal person, $B_{rational}$=.295, SE=.065, t=4.53, $P < .001$, $\eta_p^{2=}$.080; $B_{reasonable}$=.189, SE=.066, t=4.53, P=.005, $\eta_p^{2=}$.033. Notably, for communion, only reasonable and not rational scores significantly contributed to stereotype ascription for an ideal person, $B_{rational}$=.073, SE=.059, t=1.23, P=.219; $B_{reasonable}$=.284, SE=.066, t=4.33, $P < .001$, $\eta_p^{2=}$.074. We observed a similar result for selfishness, with only reasonable and not rational scores significantly contributing to stereotype ascription for an ideal person, $B_{rational}$=.081, SE=.068, t=1.20, P=.233; $B_{reasonable}$=.383, SE=.070, t=5.44, $P < .001$, $\eta_p^{2=}$.111. We did not observe significant interactions between rational and reasonable scores for any of the stereotype scores.

Study 5

Study 5a. To control for scale direction effects, half of Study 5a participants (n=133) provided the same information for *unreasonable* and *irrational* persons. Consistent with main results, unreasonable people (M=3.08, *95% CI*$_{bootsrapped}$ [2.59; 3.60]) were expected to contribute 33% less than irrational people (M=6.22, *95% CI*$_{bootsrapped}$ [5.64; 6.82]), t(265)=10.25, $p < .001$, $\eta_p^{2=}$.284. As Study 2a methods indicate, we also varied the target, asking participants to report on

expected choice for agents (“how much would a rational person give?”) vs. actions (“what would be the rational amount to give?”). The difference between implicit norms of rational vs. reasonable choice did not depend on agent-based (reasonable vs. rational agent) or action-based (reasonable vs. rational amount) framing, *F*s < 1.28, *p*s > .250.

We conducted between-subjects t-tests to compare perceptions of *rational* vs. *irrational* and *reasonable* vs. *unreasonable* others. To correct for unequal variance, t-tests followed *Wald's* t-test approach. Participants thought *irrational* (M=5.46, $95\%\ CI_{bootstrapped}$ [4.71; 6.24]) vs. *rational* people (M=3.51, $95\%\ CI_{bootstrapped}$ [3.10; 3.92]) would give more in Player A’s role, $t(df$=214.98)=4.23, $p < .001$. Similarly, expectations for irrational amounts (M=7.01, $95\%\ CI_{bootstrapped}$ [6.24; 7.66]) were significantly smaller than expectations for *rational* amounts (M=3.62, $95\%\ CI_{bootstrapped}$ [3.27; 3.99]), $t(df$=185.94)=8.53, $p < .001$.

A similar analysis suggested *reasonable* people (M=4.30, $95\%\ CI_{bootstrapped}$ [4.00; 4.58]) would give more than *unreasonable* people (M=2.16, $95\%\ CI_{bootstrapped}$ [1.53; 2.87]) in Player A’s role, $t(df$=195.46)=6.06, $p < .001$. However, expectations for *reasonable* amounts (M=4.14, $95\%\ CI_{bootstrapped}$ [3.87; 4.41]) were not significantly greater from expectations for *unreasonable* amounts (M=4.06, $95\%\ CI_{bootstrapped}$ [3.87; 4.41]), $t(df$=162.80)=.22, $p > .250$.

Study 5b-5c. In Studies 5b-5c, for exploratory purposes we examined the extent to which participants thought the characteristics of reasonable and rational applied to themselves. We also used these individual differences in self-ratings of reasonableness and rationality for predictions of personal donations on the Dictator Game. We expected participants who rated themselves as more reasonable vs. rational to predict higher donations on the game. Since each participant rated themselves on the characteristics of both reasonable and rational, we performed a within-subjects General Linear Model to account for the dependence between these two measurements. Participants rated themselves as significantly more reasonable (M=4.09, $95\%\ CI_{bootstrapped}$ [4.05; 4.13]) than rational (M=4.03, $95\%\ CI_{bootstrapped}$ [3.98; 4.08]), $F(1, 985)$=9.27, p=.002, $\eta_p^{2=}$.009. Notably, this was a very small effect. Furthermore, participants who rated themselves as more reasonable predicted they would make higher donations as Player A than those who rated themselves as less reasonable, β=.10, t (df=1)=2.51, p=.012. There was no significant effect for self-ratings of rationality leading to lower contributions, β=-.06, t (df=1)=1.49, p=.136.

In Study 5c, we found no significant difference in self-ascriptions of being rational (M=3.80, $95\%\ CI_{bootstrapped}$ [3.69; 3.91]) vs. reasonable (M=3.86, $95\%\ CI_{bootstrapped}$ [3.74; 3.98]) among university students, F(1,206)=.72, $p > .250$. Replicating Study 5a, students who rated themselves as more reasonable predicted they would make higher donations as Player A than those who rated themselves as less reasonable, β=.33, t (df=1)=4.96, $p < .001$. Furthermore, students who rated themselves as more rational predicted they would make lower donations as Player A than those who rated themselves as less rational, β=-.24, t (df=1)=3.62, $p < .001$.

Study 6

Principal component analyses indicated that responses to measures of agency, communion, and selfishness reduced to two dimensions, with the first factor (37.41% variance) characterized by communion and lack of selfishness, the second factor (20.92% variance) characterized by

agency. To remain consistent with prior scholarship (CITE), we used the average scores of warmth, competence, as well as the selfishness score for subsequent analyses.

When examining relative attribution of communion as compared to agency, participants attributed reasonable people relatively more communion than rational people, R^2=.050, F(1, 511)=26.97, $p < .001$, and relatively lower agency than rational people, R^2=.032, F(1, 511)=16.77, $p < .001$, replicating Study 1.

Greater attribution of communion among reasonable vs. rational agents was associated with greater contributions in a Dictator Game, r=.24, $p < .001$, whereas the reverse was the case for agency, r=-.15, $p < .001$. Moreover, the difference in contributions by reasonable vs. rational agents was statistically mediated by a tendency to view reasonable agents as more communal and less agentic compared to rational agents, as indicated by results of a mediation analysis with bootstrapping (2000 resamples). Specifically, simultaneously entering agency and communion scores as additional predictors into a linear regression revealed significant indirect effects of agency, β=.041, Z=3.158, p=.016, 95% *CI* [0.02, 0.07]. and communion, β=.068, Z=4.079, $p <$ .001, 95% *CI* [0.37, 0.11].

Study 7

Participants were significantly more likely to rate themselves as reasonable (M=4.19, SD=.70) than rational (M=4.12, SD=.75), t(1113)=3.74, $p < .001$. Self-ratings of rationality and reasonableness were not significantly influenced by condition-specific recall on the first task, F(1,1112)=.42, *ns*. Greater offers in the Dictator Game were associated with a greater likelihood of rating oneself as reasonable rather than rational, offer X self-rating type interaction, F(1,1112)=9.67, p=.002. To unpack this interaction, we performed simple slope analyses, revealing significantly greater contributions when participants rated themselves as more reasonable, B=.029, SE=.011, t=2.707, p=.008, but not more rational, B=.0004, SE=.011, t=.034, *ns.*

Study 8

We estimated a multi-level model with binomial choice and game type nested within participants. Participants favored a rational over reasonable agent to act on their behalf, *Wald* $\chi^{2=}$39.40 (df=1), $p < .001$. Conversely, participants favored a reasonable over rational agent for the other parties involved in a dilemma, *Wald* $\chi^{2=}$21.63 (df=1), $p < .001$. This effect was uniform across economic games, choice type X game interaction, *Wald* $\chi^{2=}$0.90 (df=2), $p > .250$.

We also assessed self-evaluations of reasonable and rational characteristics, which we entered in the multilevel model as additional predictors of choice type. Participants who rated themselves as more reasonable favored reasonable over rational agents, *Wald* $\chi^{2=}$9.31 (df=1), p=.002, whereas participants who rated themselves as more rational favored rational over reasonable agents, *Wald* $\chi^{2=}$7.96 (df=1), p=.005. The dissociation of preference for rational vs. reasonable agents for self vs. the other parties were particularly pronounced for participants who viewed themselves as more reasonable, *Wald* $\chi^{2=}$7.33 (df=1), p=.007.

As reported in the main text, participants expected rational agents to withdraw 12% more from the common pool as compared to reasonable agents in the Commons Dilemma, $t(305)=5.27$, $p < .001$, and to select a selfish (vs. prosocial) option in a Prisoner's dilemma, $\chi^{2=}7.04$ (df=1), p=.008, Cramer's V=.14. For both dilemmas, results were moderated by attributions of selfishness to rational vs. reasonable persons, Commons: $F(1,304)=7.05$, p=.008; Prisoner's: *Wald* χ^2 (df=1)= 20.24, $p < .001$. Specifically, the relative preference to attribute selfishness to rational vs. reasonable agents resulted in a greater expectation that rational agents would take more in a commons dilemma and choose a selfish option in the prisoner's dilemma.

We observed similar interaction results for attribution of agency to rational vs. reasonable persons, Commons: $F(1,304)=7.17$, p=.008; Prisoner's: *Wald* χ^2 (df=1)= 15.92, $p < .001$. Moreover, we observed the opposite interaction effect for attributions of communion, Commons: $F(1,304)=2.25$, p=.066, Prisoner's: *Wald* χ^2 (df=1)= 25.03, $p < .001$.

Finally, we examined self-evaluations of reasonable and rational characteristics, entering them in the multilevel model as additional predictors of choice type. Participants who rated themselves as more reasonable favored reasonable over rational agents, *Wald* $\chi^{2=}9.31$ (df=1), p=.002, whereas participants who rated themselves as more rational favored rational over reasonable agents, *Wald* $\chi^{2=}7.96$ (df=1), p=.005. Furthermore, the dissociation of preference for rational vs. reasonable agents for self vs. the other parties were particularly pronounced for participants who viewed themselves as more reasonable, *Wald* $\chi^{2=}7.33$ (df=1), p=.007.

Study 9

Preference for rational vs. reasonable agent to act on behalf of oneself vs. one's opponent was more pronounced in interpersonal transactions involving a legal dispute and a negotiation, type X vignette interaction, *Wald* $\chi^{2=}10.74$ (df=2), p=.005. In the standout scenario, the work-scheduling context, participants preferred a reasonable work-shift manager both if they imagined they were the business owner and if they imagined they were a subordinate. However, as Fig. 4 indicates, even within this scenario a reasonable (vs. rational) person is *more* preferred as a manager by participants who imagined themselves in the role of subordinates (compared to owners), *Wald* $\chi^{2=}22.73$ (df=1), $p < .001$. In the *judge* scenario, 67% of participants favored a reasonable (vs. rational) judge, p (binomial test) < .001, consistent with the idea that one would pick a reasonable option when the intuitive norm of reasonableness concerns socially-conscious considerations. Overall, these findings are consistent with emerging evidence on tactical preference of socially-conscious considerations, depending on situational goals (*52*).

Participants viewed rational (vs. reasonable) agents as more selfish, $t(289)=14.33$, $p < .001$, $\eta_p^{2=}.415$, which in turn moderated participants' choice: The more selfish participants viewed rational (vs. reasonable) agents, the more likely they were to pick a rational agent to act on their behalf and a reasonable agent to act on behalf of the other party. This observation dovetails with research on moral decision-making, which shows that people prefer partners who express moral judgments in deontic (i.e., interpersonally-considerate moral or emotions) rather than rational terms (*53*).

Study 10

First, we examined ratings of reasonableness and rationality when somebody defects vs. cooperates in a single-trial prisoner's dilemma by submitting the ratings to a 2 (attribution: rational vs. reasonable) x 2 (situation: defect vs. cooperate) within-subjects design. As the top panel of fig. S4, as well as figs. S5-S8 indicate, participants were significantly more likely to view a cooperating player as reasonable rather than rational and a defecting player as rational rather than reasonable, $F(1, 590)=57.71$, $p < .001$, η_p^2=.089. Next, we explored two-round games. Mixed-effects analysis yield a significant 2 (attribution: rational vs. reasonable) x 2 (round 1 player A: defect vs. cooperate) x 2 (round 1 player B: defect vs. cooperate) x2 (round 2 player A: defect vs. cooperate) interaction, $F(1, 7078)=107$, $p < .001$.

Evaluating perceived societal views on qualities attributed to rational compared to reasonable people (fig. S9), we observed a significant difference between ratings of individual-focused and socially-conscious qualities, $t(589)=22.25$, $p < .001$, η_p^2=.457. Specifically, individual-focused qualities were viewed as significantly more rational (reflected in positive values; M=.75, SD=1.00), $t(589)=18.23$, 95% CI [.670; .832]. Conversely, socially-conscious qualities were viewed as significantly more reasonable (reflected in negative values; M=-.68, SD=1.20), $t(589)=13.82$, 95% CI [-.775; -.588]. Notably, this pattern of results was significantly qualified by self-ratings of rationality, $F(1,587)=7.15$, p =.008, but not reasonableness, $F(1,587)=2.62$, p =.106. Simple slope analyses indicated the more participants viewed themselves as rational the less likely they were to attribute socially-conscious qualities solely to reasonable rather than rational people, B=.226, SE=.057, t=3.98, $p < .001$, η_p^2=.026.

Study 11

For asymmetric games (see fig. S4), general linear model analysis yield a significant 2 (attribution: rational vs. reasonable) x 2 (round 1: defect vs. cooperate) x 2 (round 2: defect vs. cooperate) interaction, $F(1, 739)=8.21$, $p=.004$, $\eta_p^{2=}.011$. Under conditions of unilateral cooperation on round 1, participants were equally likely to view defecting vs. cooperating on round 2 as reasonable, $F(1, 739)=49.91$, $p < .001$, $\eta_p^{2=}.063$, and rational, $F(1, 739)=104.33$, $p < .001$, $\eta_p^{2=}.124$. However, under conditions of unilateral defecting on round 1, participants viewed at as significantly more reasonable but not rational to cooperate rather than defect on round 2 after the other player cooperated on round 1, $F(1, 739)=63.37.33$, $p < .001$, $\eta_p^{2=}.079$; $F(1, 739)=1.31$, $p < .252$, respectively.

For symmetric games (see fig. S4), general linear model analysis yield a significant two-way interaction involving attribution type and round 2 action, $F(1, 733)=99.01$, $p < .001$, $\eta_p^{2=}.119$, but no significant three-way interaction (round 1 X round 2 x attribution type), $F(1, 739) < 1$. Whereas attribution of rationality did not significantly depend on the round 2 action, $F(1, 737) < 1$, attribution of reasonableness was significantly higher if one cooperated vs. defected on round 2, $F(1, 734)=85.41$, $p < .001$, $\eta_p^{2=}.104$.

Alternative accounts of the present findings

Is the distinction between reasonableness and rationality solely about fairness?

This alternative interpretation suggests that the present findings (especially those concerning impression formation and frame effects) are a product of reasonableness encompassing concern for fairness. Thus, reasonableness is a narrow construct, whereas rationality is a broader construct that is independent of fairness considerations.

This hypothesis can be refuted on several accounts:

1) Evidence concerning lay beliefs in Studies 1-3 shows that both descriptions of rational and reasonable persons include fair among the top 10 adjectives spontaneously mentioned by participants in Study 1 (see table S1). Thus, it's not about mere presence or absence of fairness but rather the broader socially-conscious considerations.
2) The "reasonableness=rationality+ fairness" hypothesis suggests the terms would show different semantic complexity. The *R*-based analyses for Study 1 (see below) show a tree-based diagram of frequencies with similar complexity in representation of rational and reasonable persons. Further, for both terms, fair is integrated as one among many frequent terms, to a similar extent. In short, when thinking of the characteristics of both rational and reasonable persons, lay people include fair.

Lay views on semantic structure of *rational person*

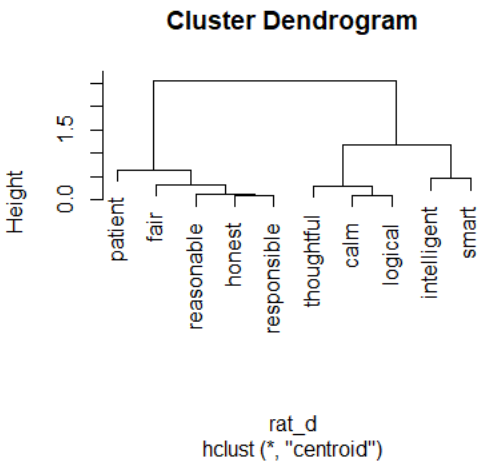

Lay views on semantic structure of *reasonable person*

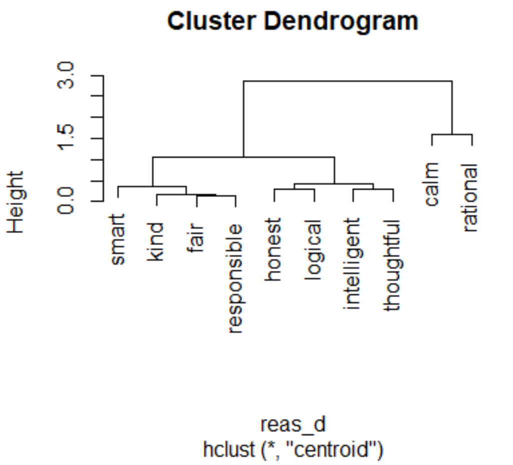

3) The “reasonableness=rationality+ fairness” hypothesis does not parsimoniously account for evidence from attribution of personality and behavioral characteristics for rational and reasonable persons in Studies 1-2 (Fig. 2 in the main text), which show that reasonable people are viewed as broadly socially-oriented, rather than only moral/fair: in addition to greater honesty/humility, they are judged as significantly higher on emotionality, extraversion and agreeableness.
4) The “reasonableness=rationality+ fairness” hypothesis cannot parsimoniously account for the attribution of satisficing to reasonable persons and attribution of maximizing behavior to rational persons. Cumulatively the evidence across these measures suggests that laypeople perceive a multi-faceted, qualitative distinction between reasonableness and rationality, consistent with our main claim, rather than simply perceiving a difference in the relative breadth of each trait.
5) As we report in the main text and the SOM, social perceptions of rational and reasonable persons uniquely contribute to social perceptions of competence/agency among the ideal person. Both concepts were independent in their alignment with the attribution of competence to an ideal person. If reasonableness were merely "rationality+fairness" it would statistically cancel out the effect of attribution of competence to an ideal person when both rational and reasonable persons are entered as predictors.
6) Evidence from content-analyses of linguistic data in Studies 3-4 shows that nouns following rational are disproportionally likely to refer to abstract/person-centered characteristics, whereas nouns following reasonable are disproportionately likely to refer to context-oriented/interdependent characteristics. Absence of fairness would not parsimoniously explain why people are disproportionately focusing on abstract/person-centric characteristics of rationality. Further, fairness was not among the key nouns differentiating reasonable from rational (see fig. S1).
7) The broader evidence in the present program of research shows that laypeople's concept of rationality is at least as narrow as their concept of reasonableness, if not more narrow. The

balance of our evidence seems to show that laypeople conceptualize rationality as involving a disposition towards unfairness (i.e., selfishness) rather than agnosticism towards fairness. In contrast, laypeople's definition of reasonableness appears much more flexible, broad and encompassing than their definition of rationality. Specifically, our evidence indicates that laypeople construe reasonableness as integrating and balancing a variety of distinct values, norms, and goals in a context-sensitive manner. Laypeople seem to construe reasonable people as flexible and able to shift their strategies and priorities in response to relevant contexts whereas they seem to construe rational individuals as more narrowly focused on maximizing their immediate preferences (see Fig. 3B).

In short, though fairness appears an important part of the concept of reasonableness, it is not sufficient to account for the pattern of results in the present program of research. Rather than viewing reasonableness as a normative judgment concerning fairness, lay views of reasonableness include a range of socially-conscious and context-integrative considerations, helping to balance what people may *actually* do with what they *ought* to do. In this way, lay views appear consistent with contractualist perspectives in political economy and philosophy (https://plato.stanford.edu/entries/contractualism/#ReaRej). Here, application of socially-conscious (contractual) considerations may contribute to a *fair society*, but do not need to be solely a product of individual-level equity.

Conversely, rationality is not merely an unfair version of reasonableness, but rather a concept of reductionist (abstraction-oriented) logical considerations of consequences (incl. preference maximization). In this way, the lay concept of rationality starts to resemble the concept of instrumental rationality in sociology and economics, often implying an individualist focus on the person/self. As such, it appears that if anything lay definitions of rationality suggest a more narrow construct of competence that focuses on optimization, instrumental calculation and preference maximization, whereas reasonableness is a broader construct that balances fairness, instrumental concerns, and moral virtues, as shown by the evidence from the economic games, and balances ideals and practical concerns, as shown by the evidence that lay people attribute satisficing goals to reasonable people.

Is the distinction between folk concepts of reasonableness and rationality solely a product of dictionary definitions?

The Oxford Dictionary (https://en.oxforddictionaries.com/definition) characterizes reasonableness and rationality as concepts concerning sound judgment. Notably, reasonableness is described as synonymous with fairness, whereas rationality is linked to logic. Thus, one may wonder whether the present results do not reflect participants' mere understanding of concepts propagated by major dictionaries.

Dictionaries are expert-driven (top-down) representations of concepts and may not correspond to how laypeople use the concepts when describing other individuals or when making economic or other mundane decisions in their lives. Though dictionary definitions are by themselves cultural products and may reflect meaning common to the lay concepts, they are not sufficient to account

for the differentiation outlined in the lay beliefs of rationality and reasonableness we demonstrate in the present research:

1) Dictionary definitions only vary in fairness and logic-related parameters, whereas analyses of spontaneous trait ascriptions (Studies 1 and 12) and cultural products (Studies 3-4) show that rational persons are more likely to be described in terms of individual-centered preferences and attributes, whereas reasonable persons are more likely to be described in terms of socially-conscious considerations (e.g., interpersonal concerns).
2) Study 2 shows that rational persons are described as maximizers, whereas reasonable persons are described as satisficers. The logic vs. fairness dictionary distinction does not parsimoniously account for these processes.
3) Evidence described above suggests that reasonable standard is not necessarily equivalent to fairness.

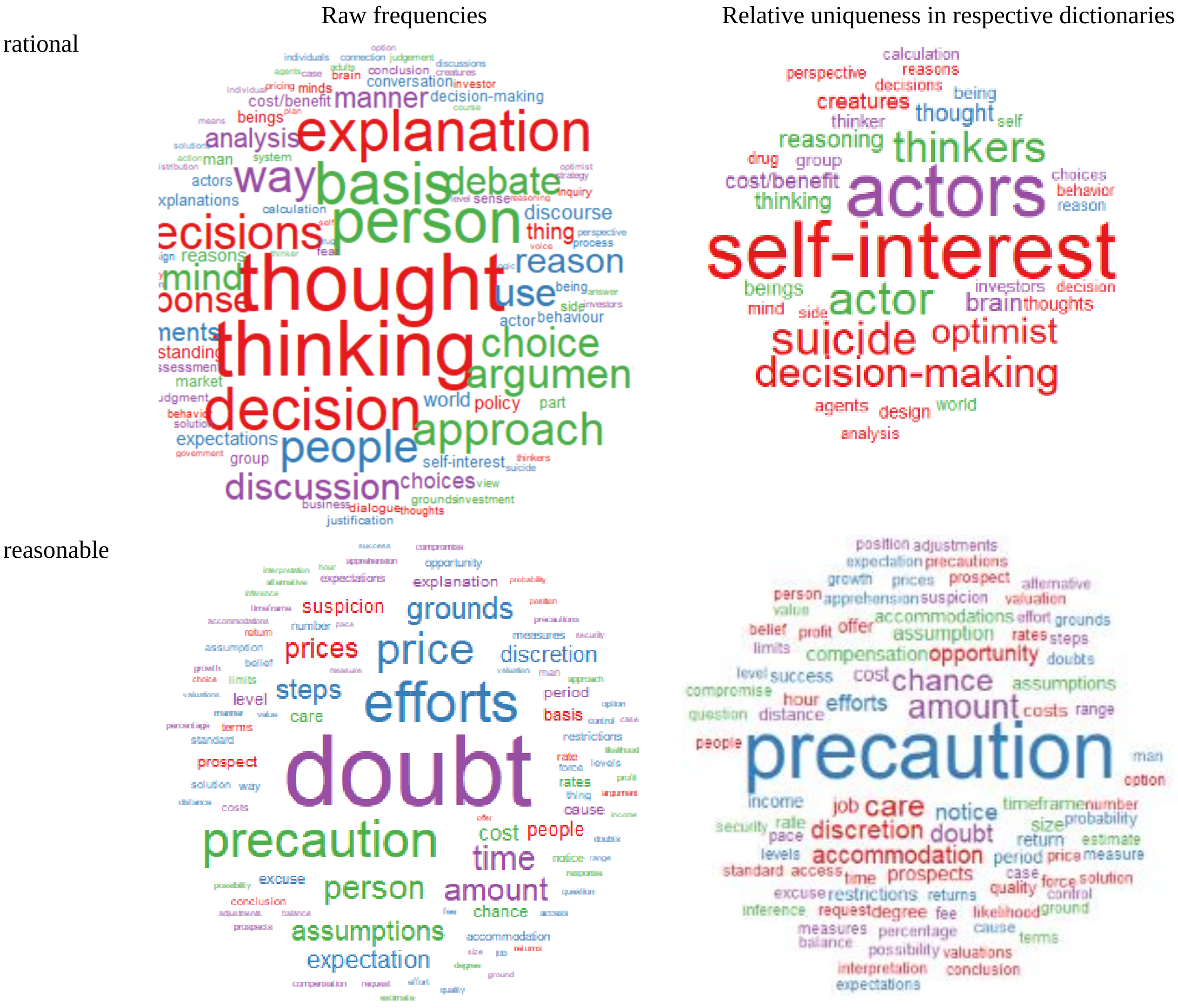

Fig. S1. Word clouds of the top 100 nouns following "rational" and "reasonable" in English language newspapers and magazines. Size of the word is based on raw frequencies (left) and relative uniqueness rank in the corpus (right).

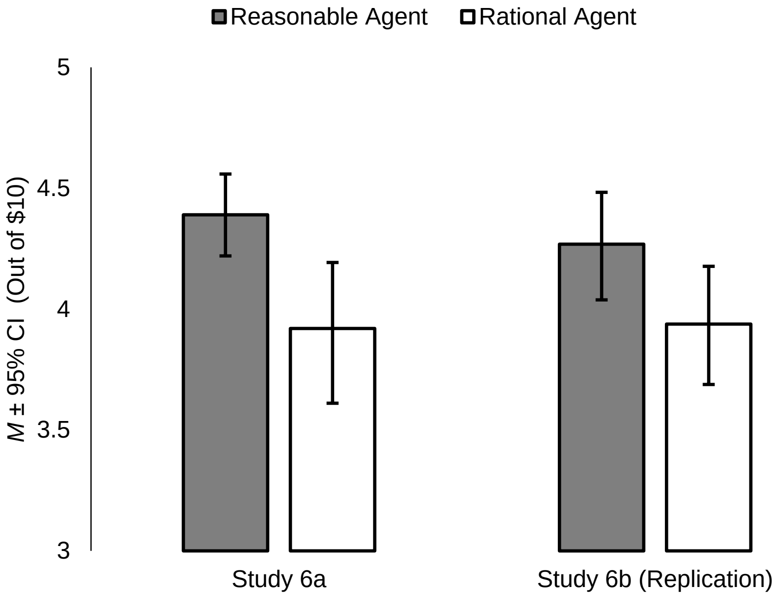

Fig. S2. Participants' contributions in the Dictator Game as reasonable versus rational agents in study 6. 95% CI = confidence interval at α = .05 obtained via bias-corrected and accelerated bootstrapping with 1000 samples.

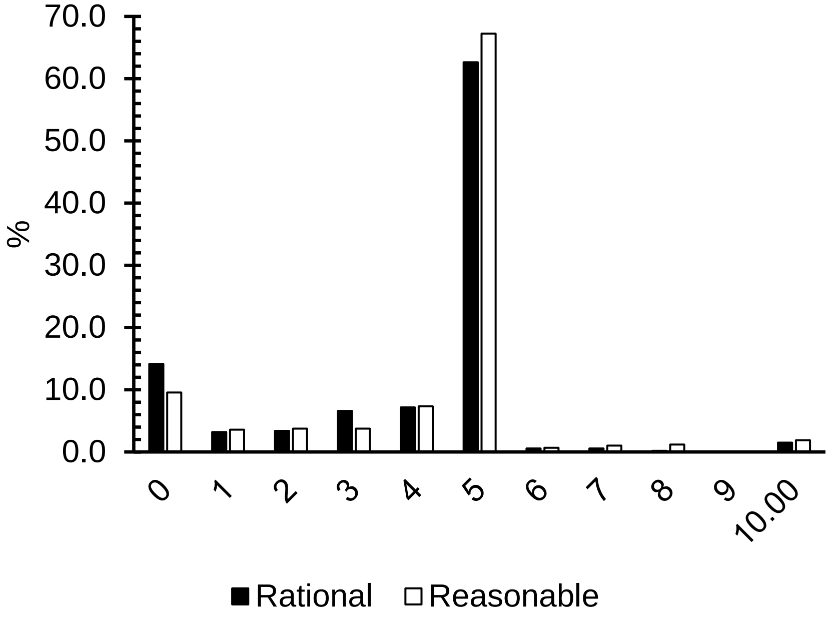

Fig. S3. Distribution of donations in the Dictator Game after reminders of rational versus reasonable experiences in study 7.

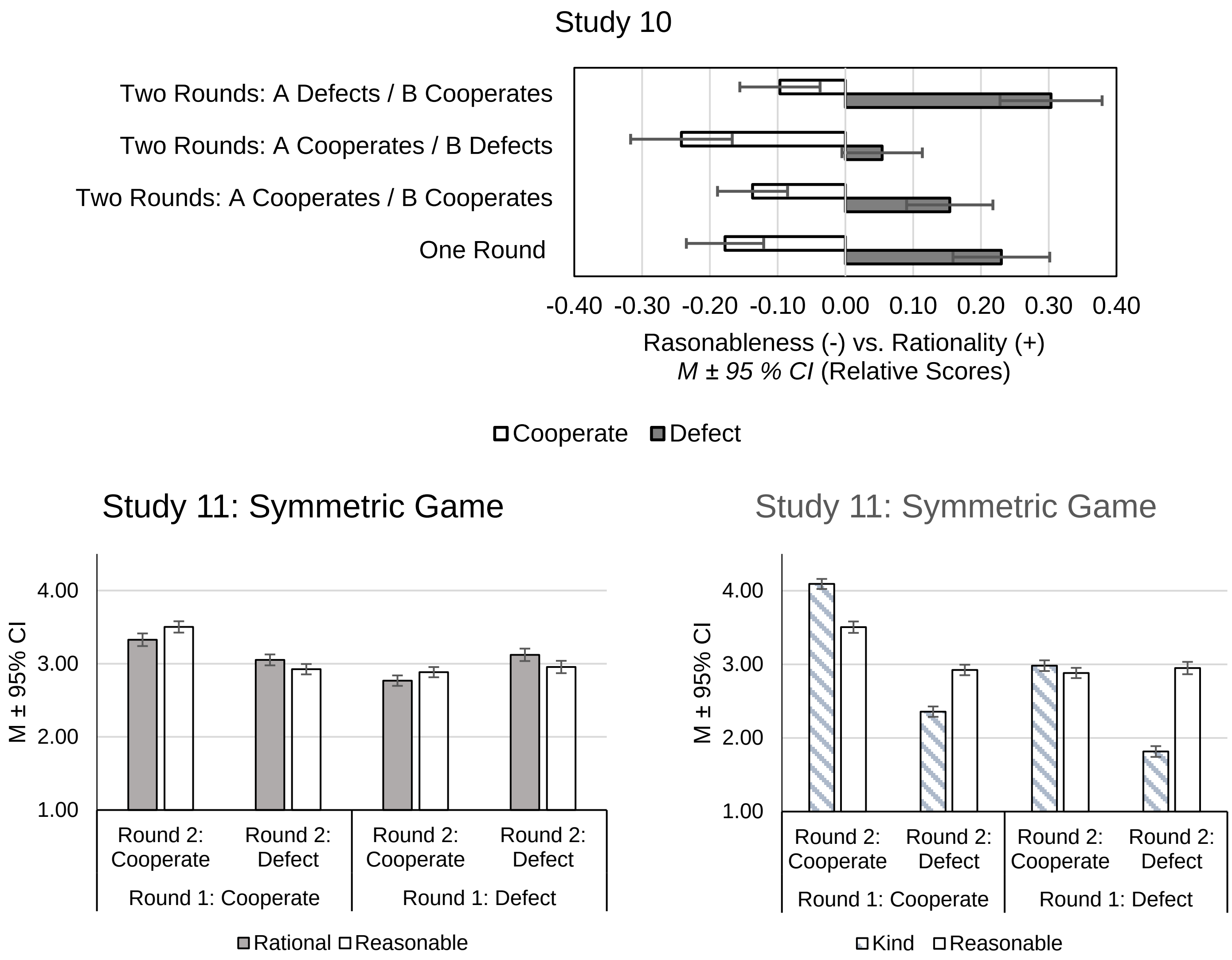

Fig. S4. Attribution of reasonableness and rationality to player A in multiround Prisoner's Dilemma in studies 10 and 11. In the top panel, for visual clarity, scores represent differences in attribution of rationality vs. reasonableness.

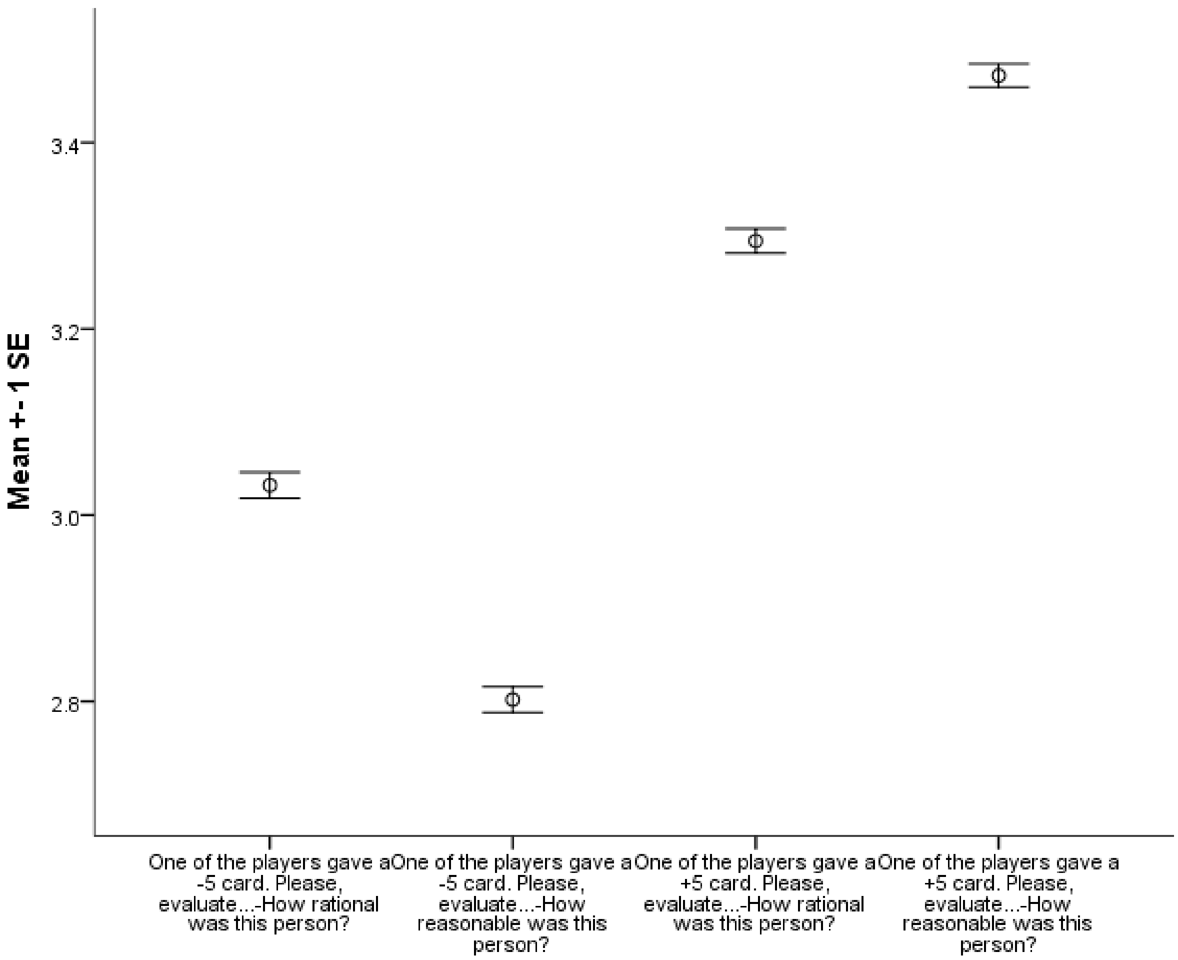

Fig. S5. Attribution of reasonableness and rationality to player A in a single-shot Prisoner's Dilemma in study 10. "-5 card"=defect/ "+5 card"=cooperate.

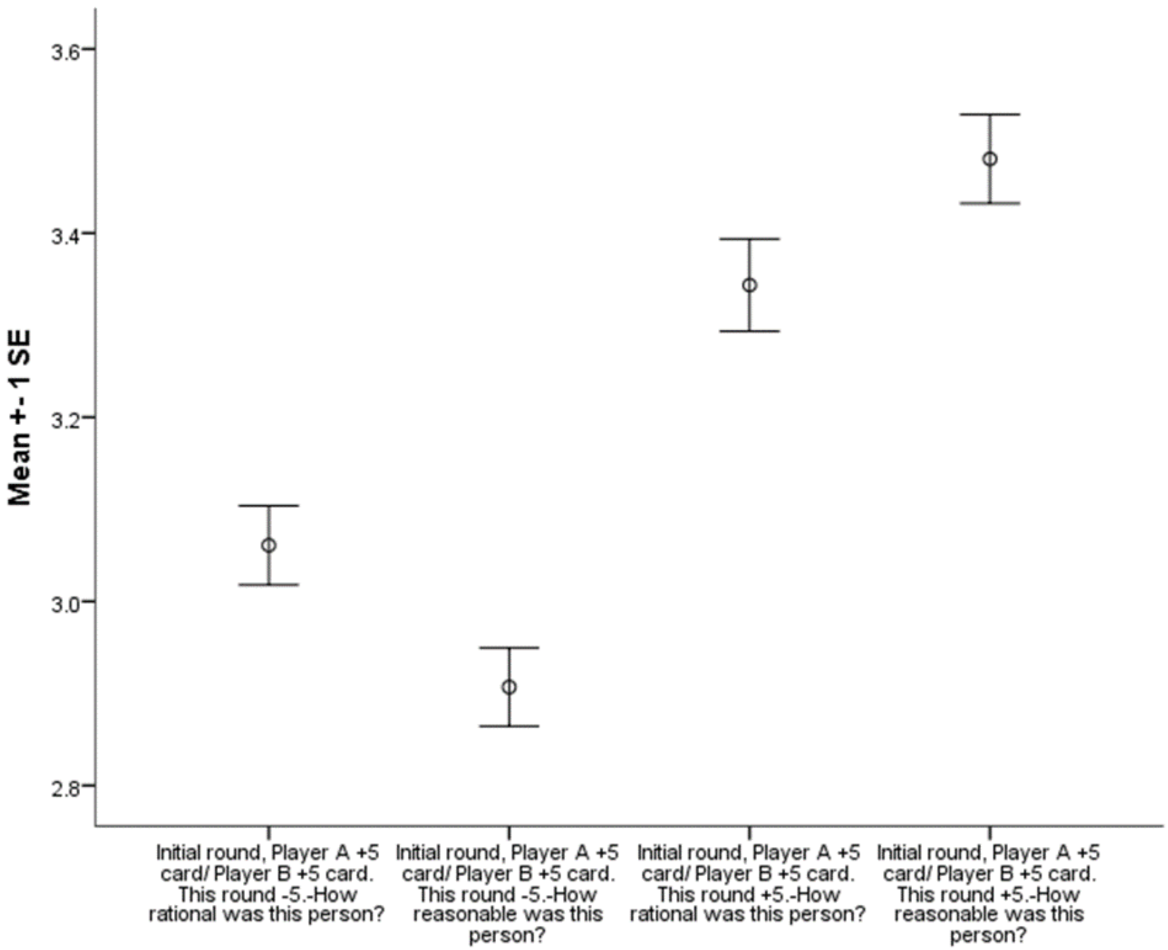

Fig. S6. Attribution of reasonableness and rationality in study 10 for cooperating and defecting players on a second round of Prisoner's Dilemmas after bilateral cooperation in the first round. "-5 card"=defect/ "+5 card"=cooperate.

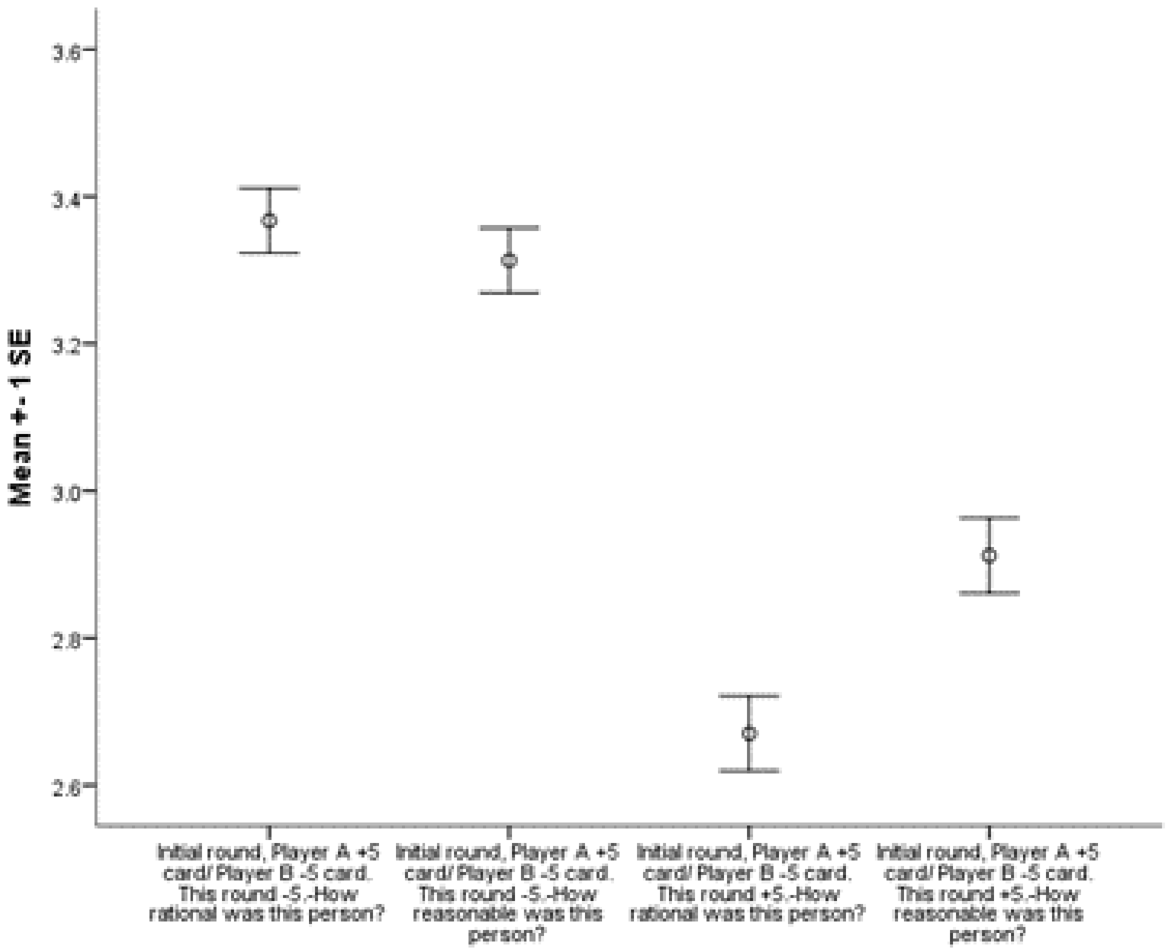

Fig. S7. Attribution of reasonableness and rationality in study 10 for cooperating and defecting players on a second round of Prisoner's Dilemmas after unilateral cooperation in the first round. "-5 card" = defect/ "+5 card" = cooperate.

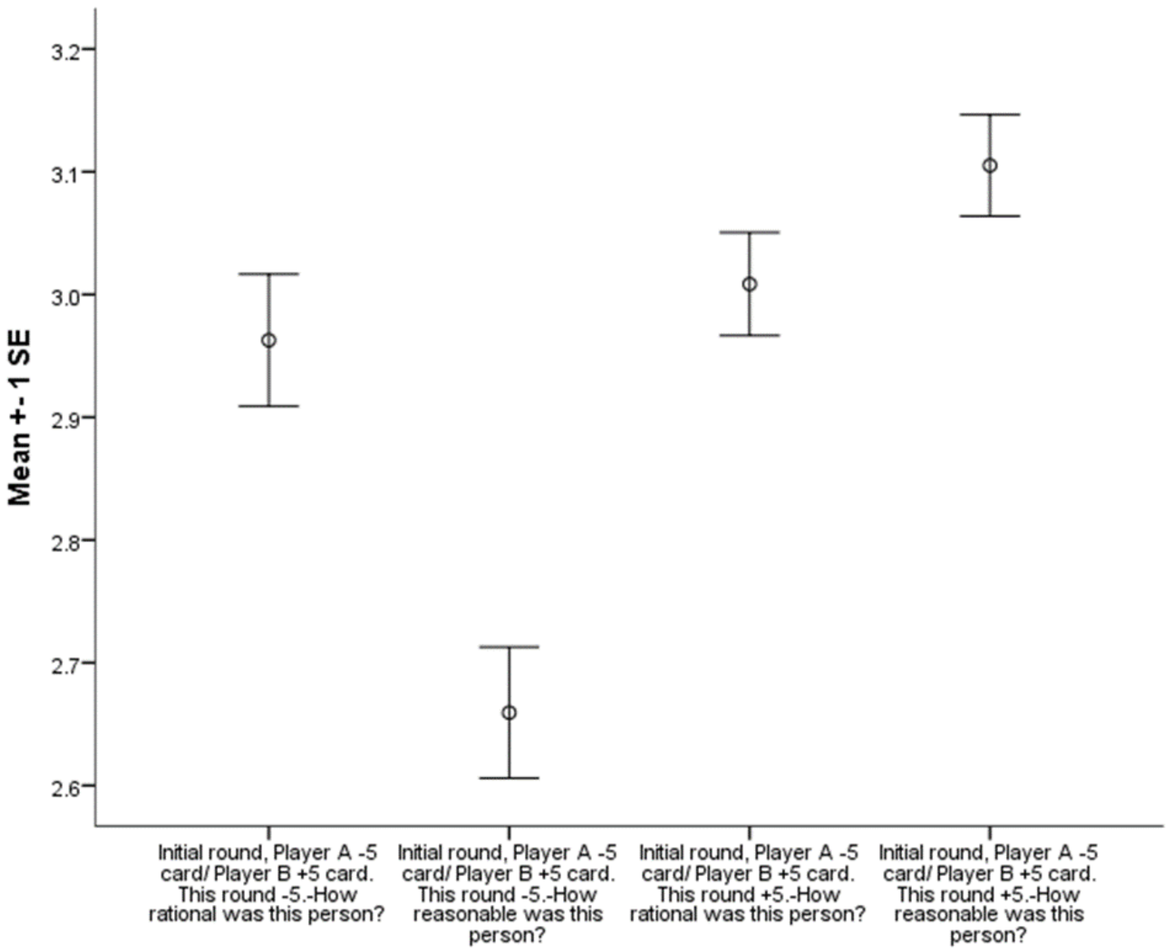

Fig. S8. Attribution of reasonableness and rationality in study 10 for cooperating and defecting players on a second round of Prisoner's Dilemmas after unilateral defecting in the first round. "-5 card" = defect/ "+5 card" = cooperate.

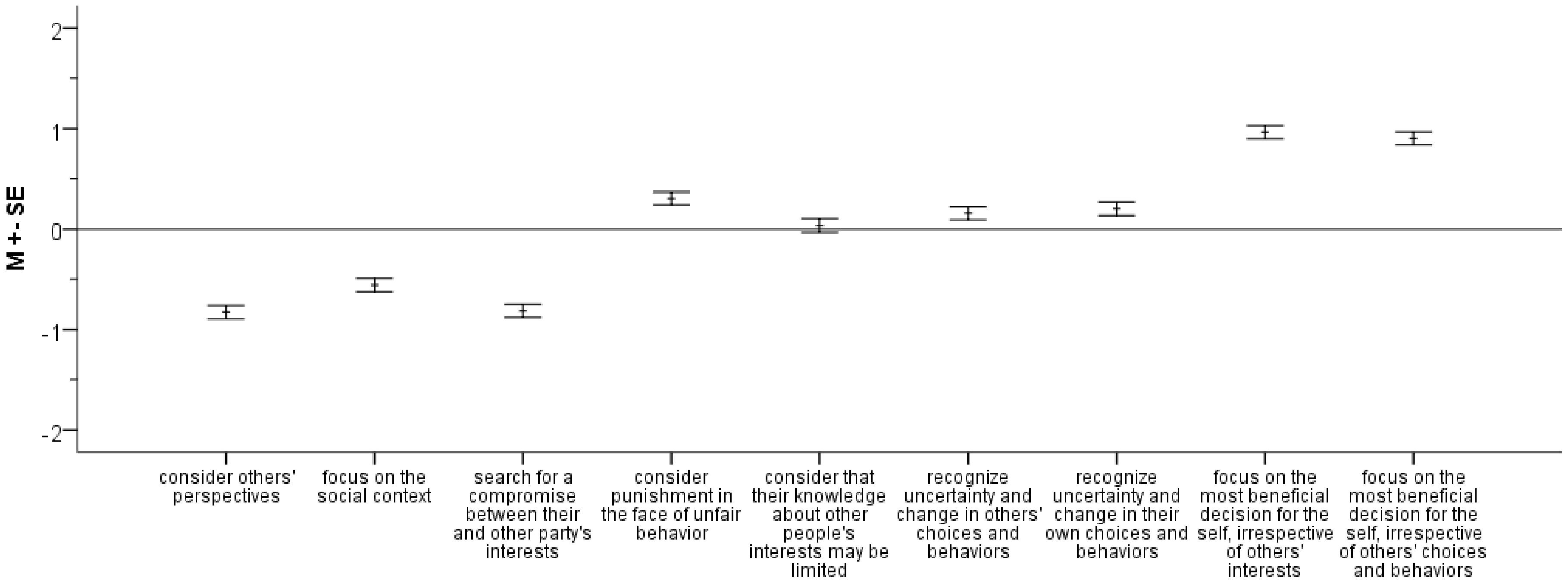

Fig. S9. Attribution of reasonableness versus rationality to different behavioral characteristics to a rational and reasonable person in study 10. Ratings of exploratory measure of behavioral characteristics in based on the wise reasoning framework (*20*). The first set of characteristics (consider others' perspectives, focus on the social context; search for a compromise between their and other party's interests; consider that their knowledge about other people's interests may be limited; recognize uncertainty and change in others' choices and behaviors; recognize uncertainty and change in their own choices and behaviors) represent common features of wise reasoning. We supplemented them with two items representing personal preference-maximization (focus on the most beneficial decision for the self, irrespective of others' interests; focus on the most beneficial decision for the self, irrespective of others' choices and behaviors) and one item concerning punishment of fairness-violation (consider punishment in the face of unfair behavior). Responses range from -2 = "More Reasonable" to 2 = "More Rational."

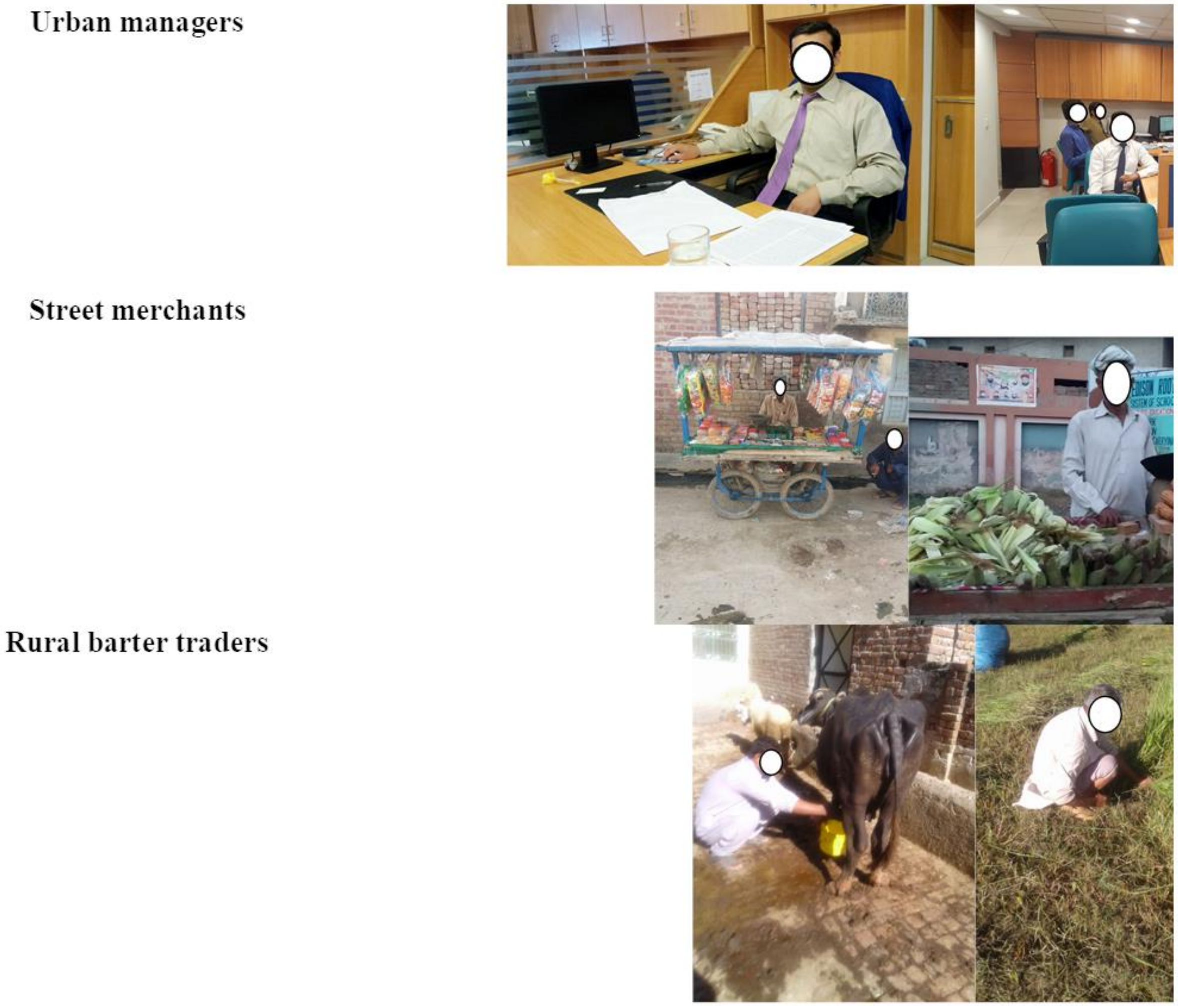

Fig. S10. Photographic depiction of survey collection sites in study 12. Pictures collected at each site. Photo Credit: Mohsin Zahid, Neuron Solutions.

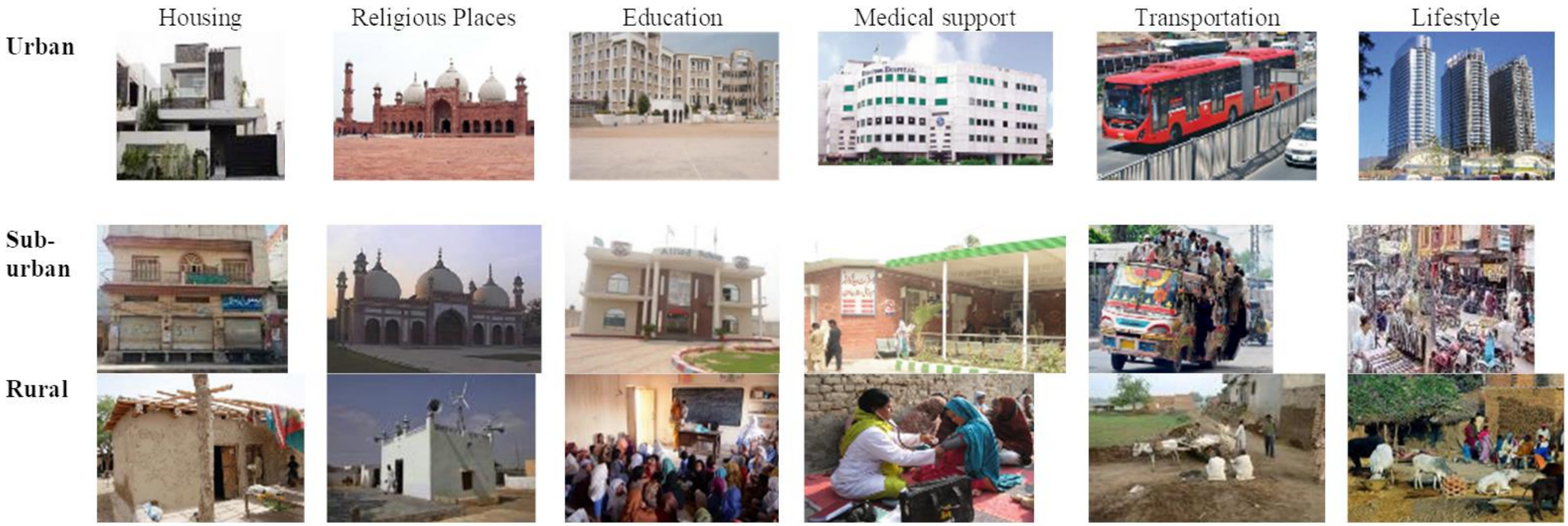

Fig. S11. Typical institutions and common places for each of the three data collection sites in Pakistan in study 12. The pictures depict the ecological landscape of each respective site in the study. Photo Credit: Mohsin Zahid, Neuron Solutions.

Table S1. Demographic information for samples used across experiments.

Study	1	2	5a	5b	5c	6a	6b	7	8	9	10	11	12
$N_{\text{Recruited}}$	289	280	690	1097	224	532	541	1187	447	294	622	815	625
N_{Valid}	239	241	548	986	207	449	515	1116	387	291	591	740	610
Age$_{\text{mean}}$ (*SD*)	37.65 (11.90)	37.10 (11.58)	36.07 (12.14)	34.87 (11.82)	20.53 (2.26)	34.99 (11.36)	36.56 (12.43)	34.51 (10.83)	35.77 (10.89)	36.71 (13.40)	35.68 (11.69)	34.86 (11.51)	32.77 (8.20)
Gender (%f)	53.4	58.6	57.8	47.6	43.4	55.5	54.3	61.2	56.9	54.8	58.8	56.7	12.3
Ethnicity (%)													
Asian-Am.	7.2	4.6	5.5	6.3	35.1	5.8	6.9	5.8	5.8	7.2	6	9.6	
African-Am.	5.9	8.8	7.2	5.4	1	5.8	6.2	6.8	7.1	5.2	7.8	9.5	
White	76.8	77.1	78.5	79.5	40	78.3	78.4	74.1	78.9	75.5	76.9	70.3	
Latino	5.1	3.8	4.6	4.7	1.5	4.0	5.4	7	5	5.2	5	5.4	
"Other"	5.1	5.8	4.2	10.4	22.4	6.1	3.1	6.3	8.2	6.9	4.3	5.2	
Income$_{\text{median}}$ *	50-75k	50-75k	35-75k	35-75k	75-100k	35-75k	35-75k	50-75k	50-75k	35-75k	50-75k	50-75k	
Education %/ years (*SD*)*													9.75 (6.42)
High school	10.9	8.8	9.7	9.2	7.8	9.4	9.5	8.7	8.4	9	11	9.5	
Some college/ vocational training	32.4	31.7	34.1	36	13.7	36.8	36	35.5	39.1	38.6	38.4	37.3	
College	43.3	47.9	40.6	38.5	45.6	37.7	37.2	39.2	36.9	37.9	35.5	36.2	
Prof. degree	2.9	2.5	2.6	3.3	7.4	3.1	2.6	1.9	1.8	1.7	2.9	2.8	
Post-grad	10.5	9.2	13	13	25.5	13	14.7	14.8	13.8	12.8	12.2	14.2	

*Note*s. Valid *N*=sample size after screening procedures (provide a response in a range between \$0-\$10, and who provided a legible response to the open-ended question concerning the recall of the task). In between-subject studies 5b-7, exclusion rates were equivalent across study conditions. * For Study 5c we used students' parental household income and highest level of parental education.

Table S2. Most frequent words (top 10%) when describing rational and reasonable persons.

Rational person			Reasonable person		
Rank	*Word*	*%*	*Rank*	*Word*	*%*
1	thoughtful	24.26	1	calm	24
2	logical	22.98	2	rational	19.11
3	calm	22.55	3	honest	13.78
4	smart	17.87	4	logical	12.89
5	intelligent	15.75	5	thoughtful	12
6	patient	8.94	6	intelligent	11.12
7	reasonable	6.38	7	smart	9.34
8	honest	5.96	8	fair	8.45
9	responsible	5.53	9	responsible	8
10	fair	4.26	10	kind	7.56
11	analytical	3.83	11	patient	6.67
11	level-headed	3.83	12	considerate	4.89
11	unemotional	3.83	13	listening	4.45
14	considerate	2.98	14	caring	4
14	deliberate	2.98	14	open-minded	4
14	mature	2.98	14	respectful	4
14	systematic	2.98	17	friendly	3.56
18	decisive	2.55	17	moral	3.56
18	event-tempered	2.55	19	happy	3.12
18	sensible	2.55	19	reliable	3.12
18	serious	2.55	19	understanding	3.12
22	educated	2.13	22	careful	2.67
22	factual	2.13	22	humble	2.67

Appendix

Coding scheme for human-based classification of statements as social/concrete vs. individual/abstract in Studies 1, 3-4, and 12

Process:

1. Read terms and code based on immediate conceptions of the phrase.
2. Consider alternate meanings of the terms. Revise coding.
3. If the phrase is not recognized, is not clearly understood, or appears nonsensical – refer to context examples on the corpus.byu.edu website.
4. Discussions between coders have largely focused on ensuring a common understanding of the coding scheme. Clarifying any ambiguities, and developing a systematic approach to reading and coding the terms

Social/concrete attributes

This category includes two components:

- Interdependent: implies or explicitly references other people or interpersonal relations and transactions, with a focus on pro-sociality, care, fairness, values, moral/religious concerns, perspective-taking, or other dimensions that are not primarily self-serving and involve balance of personal and others interests.
- Intertemporal (or interpersonal) uncertainty: focus on time, lack of certain information about others' behavior, and interests, consideration of limitations/limits, and prediction/forecasting (rather than certain expectation) of outcomes. Anything that is not set in stone by a predefined algorithm. Can be interpersonal or just in terms of planning or forecasting.

Individual/abstract attributes

This category includes two components:

- Person/preference-focused: person-related attributes, traits, personal benefits and transactions that would rather benefit the person than others.
- Formal logic: analytic processes, including reasoning, thinking, reflection, and math; words that imply logic, logical thinking and processing.
